# Performance Analysis of Orthogonal Pairs Designed for an Expanded Eukaryotic Genetic Code

**DOI:** 10.1371/journal.pone.0031992

**Published:** 2012-04-06

**Authors:** Sebastian Nehring, Nediljko Budisa, Birgit Wiltschi

**Affiliations:** 1 Department of Biocatalysis, Technical University of Berlin, Berlin, Germany; 2 BIOSS - Centre for Biological Signalling Studies, Albert-Ludwigs-University Freiburg, Freiburg, Germany; 3 Faculty of Biology, Albert-Ludwigs-University Freiburg, Freiburg, Germany; Cardiff University, United Kingdom

## Abstract

**Background:**

The suppression of amber stop codons with non-canonical amino acids (ncAAs) is used for the site-specific introduction of many unusual functions into proteins. Specific orthogonal aminoacyl-tRNA synthetase (o-aaRS)/amber suppressor tRNA_CUA_ pairs (o-pairs) for the incorporation of ncAAs in *S. cerevisiae* were previously selected from an *E. coli* tyrosyl-tRNA synthetase/tRNA_CUA_ mutant library. Incorporation fidelity relies on the specificity of the o-aaRSs for their ncAAs and the ability to effectively discriminate against their natural substrate Tyr or any other canonical amino acid.

**Methodology/Principal Findings:**

We used o-pairs previously developed for ncAAs carrying reactive alkyne-, azido-, or photocrosslinker side chains to suppress an amber mutant of human superoxide dismutase 1 in *S. cerevisiae*. We found worse incorporation efficiencies of the alkyne- and the photocrosslinker ncAAs than reported earlier. In our hands, amber suppression with the ncAA containing the azido group did not occur at all. In addition to the incorporation experiments in *S. cerevisiae*, we analyzed the catalytic properties of the o-aaRSs in vitro. Surprisingly, all o-aaRSs showed much higher preference for their natural substrate Tyr than for any of the tested ncAAs. While it is unclear why efficiently recognized Tyr is not inserted at amber codons, we speculate that metabolically inert ncAAs accumulate in the cell, and for this reason they are incorporated despite being weak substrates for the o-aaRSs.

**Conclusions/Significance:**

O-pairs have been developed for a whole plethora of ncAAs. However, a systematic and detailed analysis of their catalytic properties is still missing. Our study provides a comprehensive scrutiny of o-pairs developed for the site-specific incorporation of reactive ncAAs in *S. cerevisiae*. It suggests that future development of o-pairs as efficient biotechnological tools will greatly benefit from sound characterization in vivo and in vitro in parallel to monitoring intracellular ncAA levels.

## Introduction

Protein engineering with non-canonical amino acids (ncAAs) that are not encoded by the standard genetic code has gained much attention in the recent years. The approach is particularly interesting because it provides for the introduction of unusual functions into target proteins directly by ribosomal translation. Especially the incorporation of amino acid analogs bearing azide or alkyne side chains for subsequent bioorthogonal copper(I)-catalyzed [3+2]-cycloaddition [1] with alkyne-, or azido-ligands, respectively, is a valuable technique for the artificial post-translational modification of proteins. We and others have successfully used the methionine analogs azidohomoalanine, azidonorleucine, and homopropargylglycine [2], [3], [4] for the global substitution of Met residues in target proteins and subsequent orthogonal conjugation with fluorescent dyes, biotin, sugars, or PEG [5], [6], [7], [8], [9], [10], [11]. Quasi site-specific incorporation and conjugation, however, is only possible if the target protein contains a single Met.

Genuine site-specific incorporation of ncAAs is feasible at in-frame stop codons. Furter first demonstrated the introduction of *p*-fluoro-L-phenylalanine (pFF) at an amber stop codon *in vivo* by a “nonessential heterologous tRNA/synthetase pair” [12]. To specifically decode the amber stop codon with pFF, he chose the yeast phenylalanyl-tRNA synthetase (yPheRS) that is specific for Phe yet naturally tolerates pFF as a substrate. He introduced the yPheRS together with a compatible amber suppressor tRNA_CUA_^Phe^ into a Phe-auxotrophic *E. coli* strain harboring an endogenous PheRS mutant with greatly reduced affinity for pFF [12]. Since the identity elements of PheRS/tRNA^Phe^ pairs differ in *S. cerevisiae* and *E. coli* [13], there is little cross-species aminoacylation, i.e., the pairs are orthogonal. However, the yPheRS did not have exclusive substrate specificity for pFF, therefore, the Phe-auxotrophy of the host was required to perform the incorporation in the presence of low amounts of Phe and excess pFF. This approach ensured that Phe was still incorporated at Phe codons while predominantly pFF appeared at the position of the amber stop codon.

**Figure 1:**
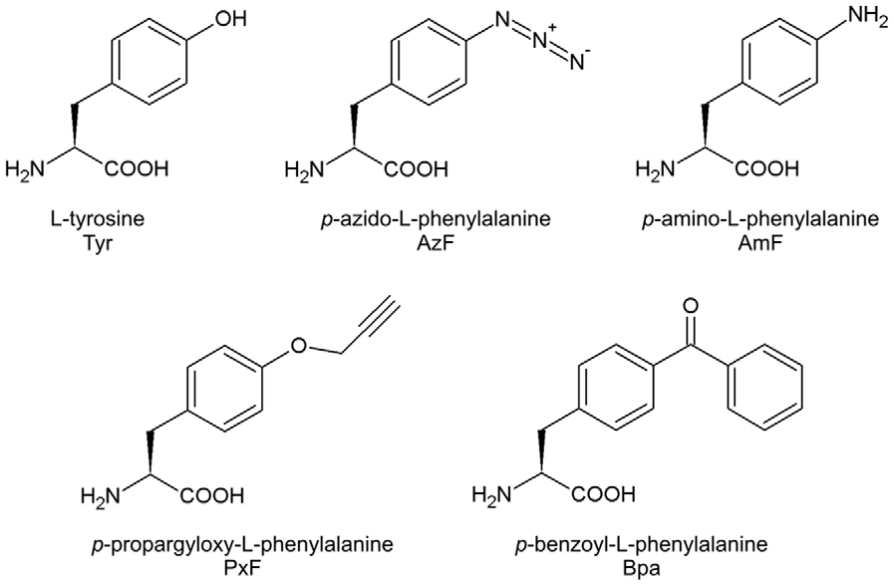
Structures of tyrosine and its analogs used in this study. Structures, names and abbreviations are shown.

Schultz and co-workers further developed the concept of orthogonal aminoacyl-tRNA synthetase/suppressor tRNA_CUA_ pairs (o-pairs) and devised an efficient screening system for the selection of orthogonal mutant aaRSs (o-aaRSs) with novel specificities for ncAAs [14], [15]. The orthogonality is achieved by importing aaRSs together with appropriate suppressor tRNAs from distantly related organisms into the host, e.g., *E. coli* components in yeast. O-pairs are paramount with respect to site specificity as they allow the incorporation of an ncAA exactly at the position of an in-frame amber stop codon. During the last decade, the Schultz group and others established o-pairs for the incorporation of a vast number of mostly Tyr analogs in different expression hosts [16], . Tyr analogs with reactive side chains, such as *p*-azido-L-phenylalanine (AzF), or *p*-propargyloxy-L-phenylalanine (PxF; Figure 1) were successfully incorporated into target proteins at amber codons and used for bioorthogonal conjugation. Recently, a new generation of o-pairs has been developed that derive from naturally occurring pyrrolysl-tRNA synthetase (PylRS)/tRNA_CUA_ pairs from methanogenic archaea and allow the incorporation of lysine analogs at in-frame amber codons (see [15] and references therein).

As outlined above, Furter used a Phe-specific orthogonal yPheRS with a natural tolerance for pFF as the substrate, in combination with a host PheRS mutant that was inefficient for pFF recognition. The Schultz approach, however, employs ncAA-specific o-aaRSs derived from tyrosyl-tRNA synthetase (TyrRS), or PylRS by directed evolution of specific amino acid residues in the substrate binding pocket [17], [18], [24]. As such, the o-aaRS/tRNA_CUA_ pairs represent autonomous decoding units for amber stop codons with a particular ncAA, that neither cross-react with host tRNAs, nor with host aaRSs. The fidelity of the system relies on the inability of the o-aaRS to charge its cognate tRNA_CUA_ with the natural substrate, Tyr, or any other of the canonical amino acids.

Despite the vast number of o-pairs that have been described so far, a systematic analysis of their catalytic properties has not yet been performed. In order to curtail this evidence gap we decided to perform a comprehensive characterization of o-pairs for the incorporation of ncAAs with reactive side chains at amber codons in yeast. The yeast *S. cerevisiae* is superior to bacterial expression hosts such as *E. coli* for the expression of integral membrane proteins and protein complexes, or for protein secretion [25], [26]. Using appropriate AzRS/tRNA_CUA_ or PxRS/tRNA_CUA_ pairs we intended to functionalize a target protein in the yeast with reactive handles by incorporation of AzF or PxF, respectively. We also included the o-pair for the photocrosslinker, *p*-benzoyl-L-phenylalanine (Bpa; Figure 1) in our study. The expansion of the genetic code with these Tyr analogs in yeast has attracted much attention [18], however, we obtained only minute amounts of target protein labeled with PxF and Bpa while AzF was not incorporated at all. In order to systematically analyze the reason for the poor incorporation, we expressed the corresponding o-aaRSs in *E. coli*, purified them to homogeneity and analyzed their catalytic activities. We found that under our assay conditions, none of the ncAAs was demonstrably activated while Tyr was recognized by all of them. We speculate that ncAAs that are poor substrates for the o-aaRSs can still be incorporated at amber codons if they accumulate in the cell due to metabolic inertness.

## Results

### Incorporation of tyrosine analogs into an amber mutant of hSOD1 in *S. cerevisiae*

For our performance analysis of o-pairs in yeast, we first intended to reproduce the site-specific introduction of azido or alkyne groups into a target protein in *S. cerevisiae* as described earlier [18]. To achieve this, we reconstructed the expression vectors pAz1/tRNA_CUA_ and pAz6/tRNA_CUA_ (Figure S1) for incorporation of AzF and PxF (Figure 1), respectively, as described in the relevant literature [18], [19], [27], [28]. Though carefully reconstructed, our expression constructs might nevertheless have deviated from the original vectors in some unrecognized aspect. Thus, we expanded our o-pair vector collection with the original plasmids pAz3/3SUP-tRNA_CUA_ [29] and pPR1/3SUP-tRNA_CUA_ [29], which were a kind gift by P.G. Schultz . The o-pair for the photo-crosslinker Bpa (Figure 1) had been applied successfully in yeast [18], [29], [30], [31], [32]. For this reason, we used pBpa/tRNA_CUA_ [18] (kindly provided by S. Hahn and P.G. Schultz) as a positive control together with pTyr/tRNA_CUA_ (reconstructed according to [27]). A schematic map of the expression plasmids for the o-pairs can be found in Figure S1 of the supporting information. Figure S2 shows a sequence comparison of the different o-aaRSs.

Upon incorporation of AzF into their target protein, Chin *et al.* observed the reduced form, *p*-amino-L-phenylalanine (AmF; Figure 1) by tandem mass spectrometry [18] rather than AzF. This observation was attributed to the chemical reactivity and photoinstability of the azido group during mass analysis [21]. However, *S. cerevisiae* can be used as a biocatalyst to reduce arylazides to arylamines [33], [34]. Therefore, we included AmF in our systematic studies in order to analyze whether AzRS would use AmF as a substrate.

In accordance with reports from the Schultz lab, we chose the human superoxide dismutase (hSOD1) as the target protein for analog incorporation. hSOD1 is a small, stable protein [35] that is well expressed in yeast. We first tested the expression efficiency of wild type hSOD1 from a construct similar to that of the Schultz group [18]. However, the expression of hSOD1 carrying a C-terminal hexahistidine-tag from the high copy, galactose inducible yeast-*E. coli* shuttle vector pYES2 was low and we did not obtain pure protein preparations (data not shown). For that reason, we replaced the hexahistidine-tag, which has been described as not ideally suitable for yeast [36], with a *Strep*-tag II. In addition, we exchanged the inducible *GAL1* promoter for the strong constitutive *PGK1* promoter. The strength of this promoter is comparable to that of the *TDH3* promoter [26], which Chen *et al.* used for hSOD1 expression together with an improved orthogonal system for tyrosine analogs in yeast [29]. Using this modified expression construct, we obtained pure hSOD1 in high yield (unpublished observation). For the incorporation of the amino acids shown in Figure 1, we introduced a Trp (TGG) to amber stop codon (TAG) mutation into hSOD1 at position 33. Previous reports demonstrated the permissiveness of this position for analog incorporation by amber suppression [18], [37], [38], [39], [40]. The map of the resulting expression construct, as well as the coding DNA sequence and the protein sequence of hSOD1(W33TAG) with a C-terminal *Strep*-tag II are shown in Figure S3 and Figure S4 of the supporting information.

Similar to the original study [18], we performed all Tyr analog incorporations in the *Saccharomyces cerevisiae* strain InvSC1 (see Materials and Methods for experimental details). Each of the o-pairs listed in Table 1 was introduced into InvSC1 together with the hSOD1(W33TAG) expression construct. We examined the efficiency of the amber suppression in hSOD1(W33TAG) using the wild type *E. coli* TyrRS/tRNA_CUA_ o-pair with Tyr. In order to scrutinize a potential residual affinity of AzRS3 for the natural substrate Tyr, we used Tyr together with the AzRS3/3SUP-tRNA_CUA_ pair. For comparison of the incorporation efficiencies, hSOD1(W33TAG) was expressed in the presence of AzF and three o-pairs with corresponding substrate specificity, AzRS1/tRNA_CUA_, AzRS6/tRNA_CUA_, or AzRS3/3SUP-tRNA_CUA_. The same o-pairs were also used with AmF. In addition, we wanted to introduce PxF into hSOD1(W33TAG) by the AzRS6/tRNA_CUA_ [18], or PxRS/tRNA_CUA_ [29] o-pairs. Finally, we introduced Bpa into hSOD1(W33TAG) with the corresponding BpaRS/tRNA_CUA_ o-pair.

**Table 1:** Details of the orthogonal pairs used for the in vivo incorporation of Tyr analogs into hSOD1(W33TAG).

Orthogonal pair	aaRS	Specificity	6xHis-tag on aaRS	tRNA_CUA_ copy number	tRNA_CUA_ promoter	Source
TyrRS/tRNA_CUA_	TyrRS	Tyr	none	1	internal B-box	reconstructed from [18]
AzRS1/tRNA_CUA_	AzRS1	AzF	none	1	internal B-box	reconstructed from [18]
AzRS3/3SUP-tRNA_CUA_	AzRS3	AzF	C-term.	3	improved*	[29]
AzRS6/tRNA_CUA_	AzRS6	AzF [18], PxF [19]	none	1	internal B-box	reconstructed from [18], [19]
PxRS1/3SUP-tRNA_CUA_	PxRS1	PxF	C-term.	3	improved*	[29]
BpaRS/tRNA_CUA_	BpaRS	Bpa	none	1	internal B-box	[18]

The pairs consist of *E. coli* TyrRS, or a mutant descendant, and *E. coli* amber suppressor tRNA_CUA_. The aaRSs are expressed under the strong, constitutive *ADH1* promoter (refer to Figure S1 for plasmid map details).

*The improved promoter consists of a yeast *PGK*1 promoter followed by three copies of the *E. coli* tRNA_CUA_ gene (with an internal B-box), each flanked by 55 bp upstream and 30 bp downstream sequences of the yeast *SUP4* gene [29].

**Figure 2:**
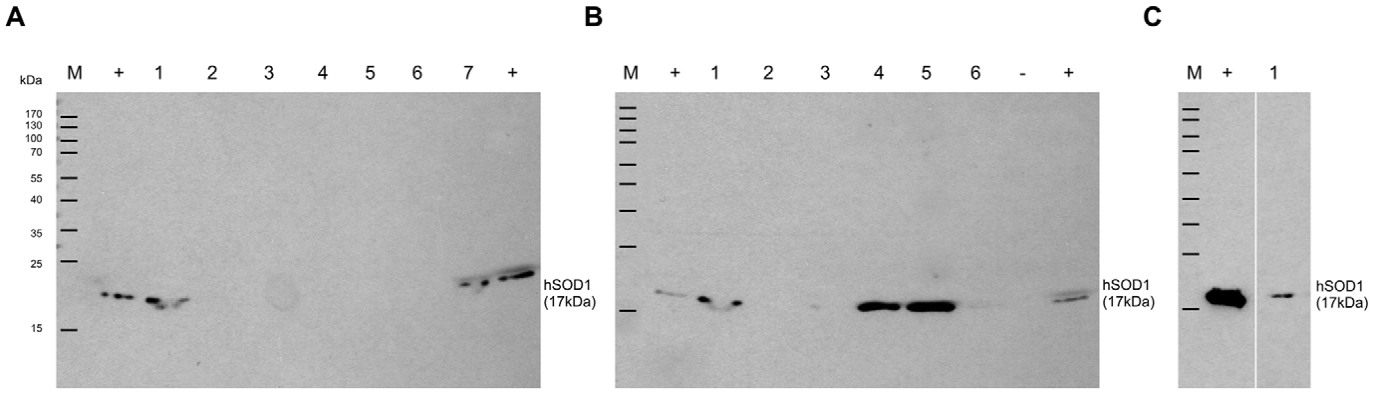
In vivo incorporation of Tyr and its analogs by amber suppression. hSOD1(W33TAG) was expressed in the presence of an o-pair and one of the analogs shown in Figure 1 as indicated below. The variant proteins were immunodetected using a monoclonal anti-*Strep*-tag II antibody (refer to the Materials and Methods section for experimental details). The average calculated molecular weight of hSOD1(W33X) is 17 kDa. (A) 1, TyrRS/tRNA_CUA_ + Tyr (replicate 1); 2, AzRS1/tRNA_CUA_ + AzF; 3, AzRS1/tRNA_CUA_ + AmF; 4, AzRS6/tRNA_CUA_ + AzF; 5, AzRS6/tRNA_CUA_ + PxF; 6, AzRS6/tRNA_CUA_ + AmF; 7, PxRS1/3SUP-tRNA_CUA_ + PxF; M; molecular weight marker; +, wild type hSOD1 with a C-terminal *Strep*-tag II. (B) 1, TyrRS/tRNA_CUA_ + Tyr (replicate 2); 2, AzRS3/3SUP-tRNA_CUA_ + AzF; 3, AzRS3/3SUP-tRNA_CUA_ + Tyr; 4, AzRS3/3SUP-tRNA_CUA_ + AmF (replicate 1); 5, AzRS3/3SUP-tRNA_CUA_ + AmF (replicate 2); 6, AzRS3/3SUP-tRNA_CUA_ + AmF (replicate 3); M and + as indicated in (A); -, empty lane. (C) 1, BpaRS/tRNA_CUA_ + Bpa; M and + as indicated in (A).

Full-length hSOD1(W33X) variants, where X denotes Tyr or an ncAA, are expressed only if the in frame amber stop codon at position 33 is efficiently suppressed by Tyr or one of its analogs shown in Figure 1. In contrast to *E. coli*, expression of heterologous proteins in *S. cerevisiae* is very often too low for detection on SDS gels in spite of the use of high copy expression vectors. Usually, the foreign protein can be immunodetected in whole cell lysates using suitable antibodies. However, we were unable to detect the full-length hSOD1(W33X) variants in whole cell lysates by immunoblotting with an anti-*Strep*-tag II antibody. We observed specific bands on the immunoblot (Figure 2) only after purification and concentration of the tagged hSOD1 variants.

We detected an hSOD1 signal on the immunoblot upon expression in the presence of the orthogonal TyrRS/tRNA_CUA_ pair together with Tyr, indicating successful amber stop codon suppression (Figure 2A and 2B, lanes 1). AzRS3/3SUP-tRNA_CUA_ in combination with Tyr caused barely detectable expression of full-length hSOD1 (Figure 2B, lane 3), which suggests good fidelity of this o-pair. However, all our attempts to incorporate AzF into hSOD1(W33TAG) with any of the o-pairs involving AzRS failed (AzRS1/tRNA_CUA_, Figure 2A, lane 2; AzRS3/3SUP-tRNA_CUA_, Figure 2B, lane 2; AzRS6/tRNA_CUA_, Figure 2A, lane 4). Surprisingly, we observed strong signals on the immunoblot in two of three hSOD1(W33TAG) expressions with the AzRS3/3SUP-tRNA_CUA_ pair and AmF (Figure 2B, lanes 4–5). The third replicate yielded only a faint band (Figure 2B, lane 6) while no bands were detected with AmF and the o-pairs involving AzRS1 and AzRS6 (Figure 2A, lanes 3 and 6). Contrary to the original study, in which a dual specificity of AzRS6 for AzF and PxF was reported [19], we obtained an hSOD1 signal on the immunoblot only when PxF was used together with PxRS1 (Figure 2A, lane 7), and not with AzRS6 (Figure 2A, lane 5). This observation may be attributed to the improved expression of the suppressor tRNA from the pPR1/3SUP-tRNA_CUA_ vector. In this construct, the suppressor tRNA is inserted between short upstream and downstream regions of the yeast *SUP4* gene. Three copies of this cassette are expressed in tandem from the strong *PGK1* promoter [29]. The same multicopy array of SUP-tRNA_CUA_ is present on the pAz3/3SUP-tRNA_CUA_ vector, which might explain the strong expression of hSOD1 in the presence of AmF with AzRS3 but not with AzRS1 or AzRS6. O-pairs with the latter aminoacyl-tRNA synthetases contain only a single copy of tRNA_CUA_ (Table 1).

As expected, we observed a clear signal for hSOD1 expression in the presence of Bpa and the BpaRS/tRNA_CUA_ o-pair (Figure 2C, lane 1).

**Figure 3:**
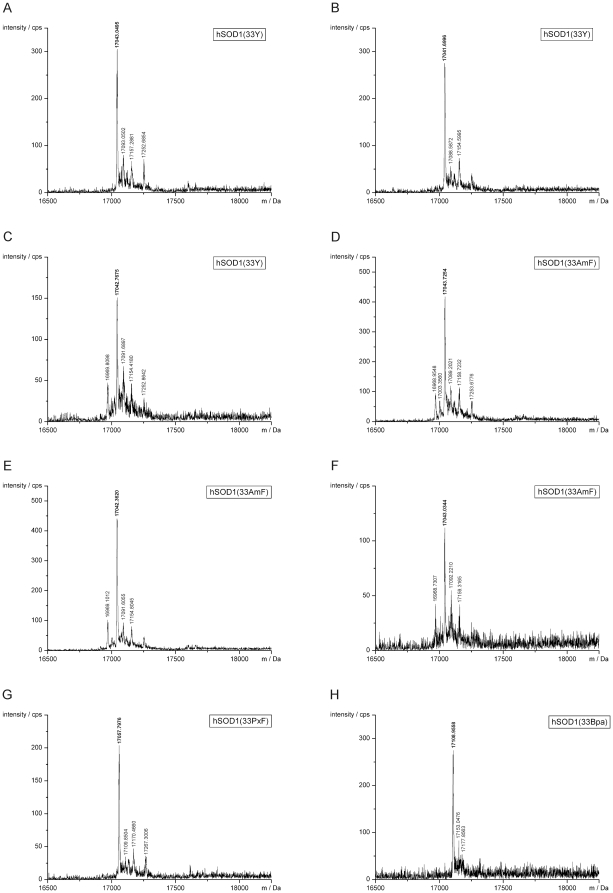
ESI-MS analyses of hSOD1 variants detected on the immunoblot in **Figure 2**. The o-aaRS which was used for amino acid incorporation is given in brackets. hSOD1(W33Y) (TyrRS), replicate 1 (A); hSOD1(W33Y) (TyrRS), replicate 2 (B); hSOD1(W33Y) (AzRS3) (C); hSOD1(W33AmF) (AzRS3), replicate 1 (D); hSOD1(W33AmF) (AzRS3), replicate 2 (E); hSOD1(W33AmF) (AzRS3), replicate 3 (F); hSOD1(W33PxF) (PxRS1) (G); hSOD1(W33Bpa) (BpaRS1) (H). For the interpretation of the main peaks (bold mass labels) refer to Table 2; none of the minor peaks (standard mass labels) corresponds to the calculated mass of hSOD1 variants containing either a canonical amino acid at position 33 or an ncAA shown in **Figure 1** (analysis details not shown).

All hSOD1 variants were purified as described in the Methods section and further analyzed by LC-ESI-MS (Figure 3), regardless whether they yielded signals on the immunoblot or not. The found mass of the tyrosine variant matched an hSOD1(W33Y) protein in which the N-terminal Met had been excised and the second amino acid, Ala, was acetylated (Table 2). Our observation is consistent with published post-translational modifications of hSOD1 produced in *S. cerevisiae* [41]. Accordingly, these post-translational modifications were taken into account for the calculation of the variant protein masses. In addition to hSOD1(W33Y), the formation of hSOD1(W33PxF) and hSOD1(W33Bpa) was unambiguously confirmed (Table 2). Although we had not detected a clear band with the AzRS3/3SUP-tRNA_CUA_ pair and Tyr on the immunoblot (Figure 2B, lane 3), the mass analysis confirmed the presence of hSOD1(W33Y) also in this sample (Table 2). However, whether AmF or rather Tyr (mass difference 1 Da) had been incorporated into hSOD1(W33TAG) using the AzRS3/3SUP-tRNA_CUA_ pair in combination with AmF could not be unambiguously deduced from our mass analyses due to low resolution (Figure 3D–F). The mass spectra of hSOD1 preparations with AzF showed only background noise signal (data not shown).

**Table 2:** ESI-MS analyses of selected hSOD1 variants.

Analog	Orthogonal pair	Calculated mass (Da)^1^	Detected mass (Da)	Δm (Da)	Interpretation	Protein per liter culture
Tyr replicate 1	TyrRS/tRNA_CUA_	17021.6943	17043.0495	21.355	+Na^+^, S-S	60 µg
Tyr replicate 2	TyrRS/tRNA_CUA_	17021.6943	17041.6996	20.005	+Na^+^, S-S	120 µg
Tyr	AzRS3/3SUP-tRNA_CUA_	17021.6943	17042.7675	21.073	+Na^+^, S-S	42 µg
AmF replicate 1	AzRS3/3SUP-tRNA_CUA_	17020.7096	17043.7254	23.016	+Na^+^	78 µg
AmF replicate 2	AzRS3/3SUP-tRNA_CUA_	17020.7096	17042.3620	21.652	+Na^+^, S-S	114 µg
AmF replicate 3	AzRS3/3SUP-tRNA_CUA_	17020.7096	17043.0344	22.325	+Na^+^	90 µg
PxF	PxRS1/3SUP-tRNA_CUA_	17059.7424	17057.7976	−1.945	S-S	96 µg
Bpa	BpaRS/tRNA_CUA_	17109.8012	17108.9558	−0.845		80 µg

Only variant proteins for which defined mass spectra were obtained are shown. The same hSOD1 variants were detected on the immunoblot in Figure 2. The corresponding ESI-MS spectra are shown in Figure 3. All hSOD1 variants were found with the N-terminal methionine cleaved off and acetylated alanine at position 2, as reported in the literature [41]. The occasionally attached sodium ions (+22.99 Da) most probably originated from the *Strep*-Tactin elution buffer which contained 150 mM NaCl. The buffer was not exchanged during sample concentration in order to avoid protein loss. In some of the protein preparations we found a known disulfide bond (S-S, −2 Da; between C57 and C146 [70]).

1 All hSOD1 masses were calculated without N-terminal methionine, acetylated alanine at position 2 and with completely reduced cysteines.

The yields of the confirmed hSOD1(W33X) variants (Table 2) ranged between approximately 42 and 120 µg per liter yeast culture. These amounts are noticeably lower than the previously published values of milligrams per liter culture [29], [42]. The protein concentrations of the variant preparations were too low to allow analysis of their purity by SDS-PAGE.

Due to the inefficient or inexistent incorporation of PxF and AzF, respectively, we analyzed the intracellular expression of their specific aaRSs by immunoblotting. The expression of AzRS1 from pAz1/tRNA_CUA_ was clearly visible, however, we could hardly detect AzRS3 and PxRS (Figure S5).

In summary, we observed suppression of the amber stop codon and hence expression of full-length hSOD1(W33X) with Tyr, Bpa, PxF, and AmF but not with AzF. The incorporation of Tyr, Bpa, and PxF into the target protein was confirmed by ESI-MS analysis. The incorporation of AmF could not be unambiguously revealed. PxF was only incorporated into the target protein with the improved PxRS1/3SUP-tRNA_CUA_ o-pair (Table 1). In our hands, the o-pairs for yeast performed less efficiently than expected although we exactly followed the published methods and used original expression constructs or reconstructed them according to previous reports.

### In vitro activation of tyrosine analogs by the different o-aaRSs

Besides the *in vivo* performance analysis, another important aim of our study was to scrutinize the amino acid activation profiles of the o-aaRSs *in vitro*. These data provide important information about the substrate binding by the o-aaRSs. We performed *in vitro* ATP-PPi exchange assays [43], [44] (see Materials and Methods for technical details) with the same o-aaRSs we had used for the *in vivo* incorporation of Tyr analogs into hSOD1(W33TAG). Basically, aminoacylation is indispensable for protein translation and occurs in a two-step process. The amino acid is first activated and then charged onto its cognate tRNA by a specific aaRS. The activation reaction consumes ATP and pyrophosphate is released. As this reaction is reversible, the amount of radioactive ATP that is formed from [^32^P]-pyrophosphate in the reverse reaction is a measure for the activation of an amino acid by an aaRS.

In order to prepare pure enzymes for the activation assay, we constructed His-tagged fusions of TyrRS, AzRS1, AzRS6, AzRS3, and BpaRS for expression in *E. coli* and subsequent purification by Ni-chelate chromatography (refer to Materials and Methods for details). The purified aaRSs were characterized by SDS-PAGE (Figure S6) and ESI-MS analysis (Table S1; Figure S7). In a first step we determined the optimal amino acid concentration range for the assay. To achieve this, we quantified the radioactive ATP produced by 1 µM TyrRS within 15 minutes with a range of Tyr concentrations (Figure S8). A steady increase in ATP formation and hence amino acid activation was found for Tyr concentrations between 5 µM and 100 µM. Tyr concentrations above 100 µM saturated the enzyme and did not further increase ATP formation.

**Figure 4:**
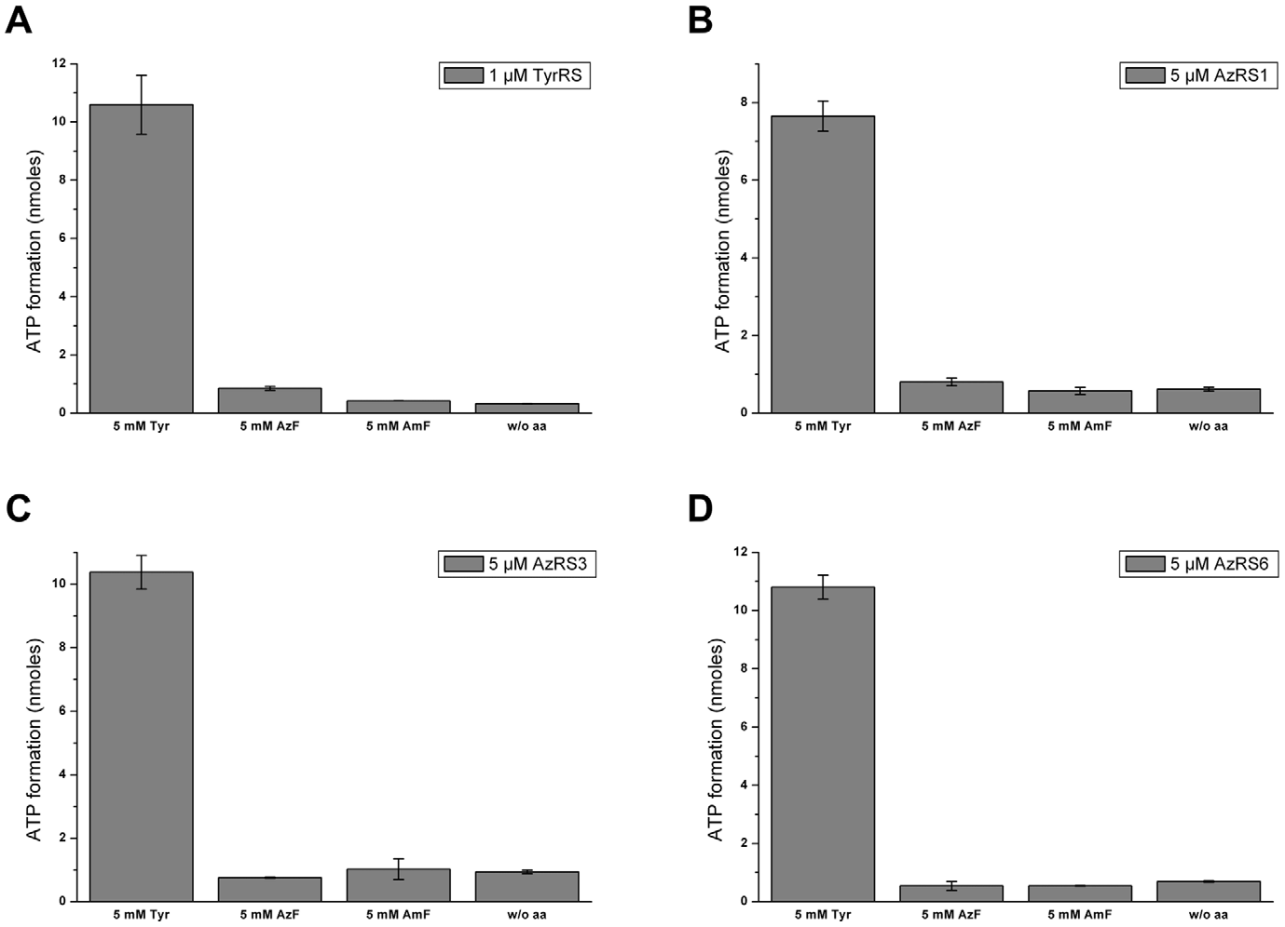
Activation of Tyr, AzF, and AmF by TyrRS and the AzRSs. Tyr, AzF and AmF were used at a concentration of 5 mM. No amino acid was added to the negative control (w/o aa). TyrRS (A) was added at 1 µM and AzRS1 (B), AzRS3 (C), and AzRS6 (D) at a concentration of 5 µM. The data for each o-aaRS were collected in one series of experiments (see Materials and Methods for details). The average of duplicate determinations is shown; the bars indicate the discrete values.

**Figure 5:**
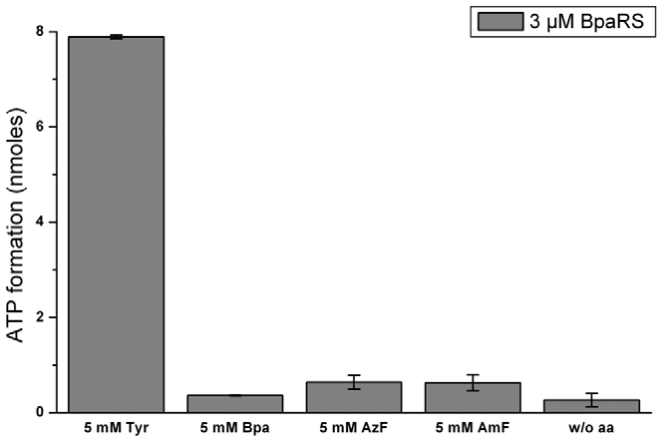
Activation of Tyr, Bpa, AzF, and AmF by BpaRS. Tyr, Bpa, AzF and AmF were used at a concentration of 5 mM. In the negative control, the amino acid was omitted (w/o aa). BpaRS was added at a concentration of 3 µM. The data were all recorded in one row of experiments and each value was determined in duplicate. The bars denote the discrete values.

Next, we assayed the activation of Tyr and the analogs AzF and AmF by TyrRS and the ncAA-specific AzRS1, AzRS3, AzRS6, and BpaRS (Figure 4 and Figure 5). With the latter enzyme, an activation assay with Bpa was also performed. We suspected the Tyr analogs to be worse substrates for their cognate aaRSs than Tyr is for the TyrRS. Therefore, the amino acids were added in excess to a final concentration of 5 mM each. 1 µM TyrRS, 5 µM of the AzRS proteins, and 3 µM BpaRS were used in the assay. Tyr and its analogs are barely water soluble, thus, the substances were dissolved in 0.01 M HCl. Accordingly, the negative control (Figure 4 and Figure 5; w/o aa) contained no amino acid but the appropriate amount of HCl.

As expected, Tyr was efficiently activated by TyrRS (Figure 4A). The activation data are in good agreement with the successful incorporation of Tyr *in vivo* (Figure 2A and 2B, lanes 1). TyrRS did not activate AzF nor AmF (Figure 4A), confirming the lack of natural tolerance for these substrates. However, we found no activation either of AzF or AmF by AzRS1, AzRS3, or AzRS6 (Figure 4B–D). On the contrary, all AzRSs activated Tyr (Figure 4B–D). Similarly, BpaRS did not activate Bpa but Tyr was efficiently recognized (Figure 5). The inactivity of AzF in the ATP-PPi exchange assay with the AzRSs coincides with our finding that this analog was not incorporated into hSOD1(W33TAG) *in vivo* (Figure 2). In contrast, hSOD1 was expressed in the presence of AmF (Figure 2B, lanes 4–6) and Bpa (Figure 2C, lane 1), yet the analogs were not activated *in vitro*.

In order to exclude that the high concentration (5 mM) of the Tyr analogs caused an inhibition of the o-aaRSs, we assayed the activation of Tyr, AzF, and AmF also in the lower concentration range (500, 50, 5, and 1 µM; Figure S9). Once again, Tyr was activated in a concentration dependent manner while we did not detect activation of AzF nor of AmF.

In summary, under our experimental conditions the o-aaRSs did not demonstrably activate the Tyr analogs but properly activated their original substrate, Tyr.

## Discussion

We evaluated the incorporation of reactive ncAAs with existing o-pairs at the in-frame amber codon of an hSOD1(W33TAG) mutant in yeast. AzF-, PxF-, and Bpa-specific o-aaRSs for the corresponding o-pairs in *S. cerevisiae* were first evolved by Chin *et al.* [18]. The same o-aaRSs were used later to construct an improved expression system in yeast [29], and to establish the amber suppression incorporation of these ncAAs in *P. pastoris* [20].

The Schultz group reported incorporation of AzF and PxF into hSOD1 in yeast using the evolved o-pairs [18] and subsequent bioorthogonal conjugation to alkyne- or azido-functionalized fluorophores [19] and PEG [42]. Becker *et al.* used the Bpa-specific o-pair to introduce Bpa site-specifically into the G-protein coupled receptor (GPCR) Ste2p in yeast. The Bpa-labeled Ste2p could then be photocrosslinked to its peptide ligand [31]. The groups of Sakmar and Wang used the AzF- and Bpa-specific o-pairs developed in yeast to incorporate these ncAAs into proteins expressed in mammalian cells [45], [46]. Different GPCRs were labeled with AzF and Bpa for subsequent dynamics measurements by Fourier-transform infrared (FTIR) difference spectroscopy [47], [48] and to analyze ligand binding [49], [50] (for a comprehensive review on the incorporation of molecular probes to study GPCRs see [51]).

Although we used original or thoroughly reconstructed tandem expression constructs for the o-pairs and followed published methods, we obtained unexpected results. In our hands, none of the three different AzRS/tRNA_CUA_ pairs (Table 1) previously described to work efficiently [18], [19], [29], [42] promoted amber suppression with AzF.

PxF and Bpa were incorporated by their cognate o-pairs in response to the amber codon at position 33 of hSOD1, though less efficiently (Table 2) than previously reported [29]. This finding indicates that the failure to incorporate AzF with the AzRS/tRNA_CUA_ o-pairs did not originate from our experimental setup.

We observed incorporation of PxF into hSOD1(W33TAG) with the tandem vector for optimized tRNA_CUA_ expression (Table 1; [29]) while the originally described vector containing only one copy of the gene and no optimized promoter (Table 1; [18]) was inefficient. This confirms the published observation [29] that three copies of tRNA_CUA_ and the optimized promoter result in improved amber stop suppression.

Surprisingly, we obtained a rather strong immunoblot signal upon expression of hSOD1(W33TAG) in the presence of AmF and the AzRS3/3SUP-tRNA_CUA_ o-pair optimized for AzF (Figure 2B, lanes 4–5). Our attempts, however, to confirm incorporation of AmF by mass analysis were inconclusive owing to a calculated mass difference of only 1 Da between AmF and Tyr. The ESI-MS produced strong signals but the resolution was too low to unambiguously distinguish between intact hSOD1(W33AmF) and hSOD1(W33Y) (see Table 2 for calculated and found masses; Figure 3D–F). Direct LC-MS/MS analysis of the trypsin-digested protein preparation was disappointing (data not shown), as we could not identify the specific peptide fragment carrying the ncAA. Yet, there is indirect evidence that we isolated an hSOD1(W33AmF) variant. The concentrations of Tyr and the other canonical amino acids in the expression medium were too low to support amber suppression with the AzRS3/3SUP-tRNA_CUA_ pair as can be concluded from the unsuccessful incorporation experiments with AzF. These observations strongly argue against the incorporation of Tyr or any other canonical amino acid in the presence of AmF.

Our finding indicates a hitherto unrecognized specificity of AzRS3 for AmF. Indeed, o-aaRSs with extended substrate tolerance have recently been described [52], [53]. These o-aaRSs accept various ncAAs (up to 18 different species [53]) as their substrates yet they retain their ability to discriminate against the 20 canonical amino acids. The tolerance of AzRS3 for AmF might occur coincidentally as described for other o-aaRSs. It may, however, have been nurtured by the chemical properties of AzF. AmF is a reduced derivative of AzF. It was observed after incorporation of AzF into different target proteins produced in various host systems (*E. coli* [54], [55], *S. cerevisiae* [18], [19], [29], [42], and mammalian cells [21]), using different purification methods (Ni-chelate chromatography [18], [19], [29], [42], [54], [55] *vs.* affinity chromatography [21]), or different mass analysis techniques (ESI-TOF [54], LC-MS/MS [18], [19], [21], [42], or multiple-reaction monitoring [29]). These observations were accredited to “the chemical reactivity and photoinstability of the azido group” [21] or interpreted as artifacts generated during mass analysis [18], [54]. e.g., by the reducing conditions used for in-gel digestion of proteins. Mass spectrometry of full length proteins does not necessarily require reducing conditions. Accordingly, Chin *et al.* did not detect decomposition of AzF to AmF by mass spectrometry of intact AzF-labeled sperm whale myoglobin isolated from *E. coli* [55]. Moreover, they purified their protein “under a red photographic light, to avoid photolysis of the aryl-azide” [55]. In contrast, Wan *et al* observed noticeable amounts of AmF in their protein preparations by ESI-TOF analysis. Most probably this was due to the reducing buffer with 10 mM DTT in which they had lysed the *E. coli* cells [54]. Whether decomposition of AzF to AmF already occurs in the cellular environment or is an artifact generated by the conditions during mass analysis, or both, has not yet been unequivocally resolved. This issue is important with respect to bioorthogonal conjugations for which the azido group is indispensable.

AmF insertion in place of AzF in yeast might originate from the direct incorporation of AmF by a dual specificity of the o-aaRS. Arylazides such as AzF can be biocatalytically reduced to the amines by yeast [33], [34]. The (partial) conversion of AzF into AmF already during the selection process of the o-aaRS could possibly lead to the selection of an enzyme with dual substrate specificity. In our experiments, AzRS3 behaved more like an AmF-specific o-aaRS since we observed full length hSOD1 only upon amber suppression with AmF and not with AzF (Figure 2B, lanes 4–6 *vs.* lane 2).

As already mentioned above, the Schultz group was able to incorporate AzF into a hSOD1(33TAG) mutant and demonstrated the bioorthogonal conjugation of the azido-functionalized protein with two different alkyne-fluorophors [19] and alkyne-PEG [42]. However, from these reports it cannot be deduced whether the AmF variant was also present in their protein preparation. They exclusively found AmF containing tryptic fragments in their LC-MS/MS analysis, most probably again due to the reducing conditions during in-gel digestion. However, they did not discuss this issue. They also performed a MALDI-TOF analysis of the intact PEGylated protein but the mass range in the presented spectrum is cut off at approximately 18500 Da [42] and, therefore, does not extend to the lower masses of the unconjugated AzF or AmF variants (calculated masses: 16671.2560 Da and 16645.2584 Da, respectively; the calculation was based on the hSOD1 sequence published by Tippmann *et al.* [40] and N-terminal methionine excision and acetylation of the alanine residue in the second position were taken into account; the calculated mass difference between the N-terminally modified and the unmodified proteins is 89 Da).

The unsatisfactory incorporation of AzF and PxF in our study might originate from inefficient expression in yeast of their respective charging enzymes, AzRS, and PxRS. Previous reports largely neglected this issue. Chen *et. al* analyzed the mRNA levels of a PxF-specific o-aaRS, however, they did not analyze the protein expression [29]. The intracellular expression of AzRS1 from our reconstructed tandem vector (Figure S1 and Methods S1) was clearly detectable by immunoblotting while we observed no or only low expression of AzRS3 (from pAz3/3SUP-tRNA_CUA_) and PxRS1 (from pPR1/3SUP-tRNA_CUA_), respectively (Figure S5). Since AzF was neither incorporated in the presence of AzRS1 nor of AzRS3, the intracellular availability of the charging enzyme does not appear to be the primary reason for the incorporation failure. The PxRS1 levels, however, could have been too low for efficient activation and charging of PxF. In order to obtain a comprehensive overview of o-pair functionality, it would be necessary to analyze not only the intracellular levels of the o-aaRSs but also those of the empty *vs.* charged tRNA_CUA_.

In order to strengthen our performance analysis of yeast o-pairs we not only conducted *in vivo* incorporation experiments with different ncAAs but also validated these findings against catalytic data collected *in vitro*. For the characterization of the catalytic properties of TyrRS, AzRS1, AzRS3, AzRS6, and BpaRS, we purified the enzymes from *E. coli* and analyzed their ability to activate different ncAA substrates *in vitro*. We could not include PxRS1 in this study since our PxF preparation contained residual Tyr as detected by mass analysis (Figure S10), which would have obscured the results of the activation assays. Unfortunately, PxF is not commercially available.

As expected, TyrRS activated only Tyr and neither AzF, nor AmF (Figure 4A). The purified o-aaRSs, however, did not activate AzF, AmF nor Bpa, while Tyr was activated by all of them (Figure 4B–D and Figure 5). Due to the fact that the activation readout was close to background at all tested analog concentrations (Figure 4B–D, Figure 5 and Figure S9), even when they were used at 5 mM, we were unable to assess the Michaelis constant K_M ncAA_ of the different enzymes. Our findings cannot be attributed to an inadequate experimental setup since under the same conditions the previously described *E. coli* Gly294PheRS mutant enzyme [56], [57], [58], [59] activated a whole set of Tyr, Phe, and Trp analogs as shown in Figure S11.

Contrary to our expectations, we found that all o-aaRSs activated preferentially Tyr (Figure 4B–D, Figure 5, and Figure S9). However, Tyr was rather inefficiently incorporated into hSOD1(W33TAG) *in vivo* by the pAz3/3SUP-tRNA_CUA_ o-pair only if the medium was supplemented with excess Tyr (Figure 2B, lane 3). Unfortunately, the Schultz group did not report the kinetic parameters of the o-aaRSs derived from *E. coli* TyrRS for the incorporation of AzF or Bpa in yeast. Thus, we can only speculate why an amino acid that is clearly activated by the o-aaRSs only appears in the target protein if it is supplied in excess in the medium (Figure 2B, lane 3). In a prototrophic yeast strain growing in minimal medium without tyrosine, the intracellular Tyr concentration varies between 0.2 mM and 3 mM during the different growth phases [60]. Our SC –Ura –Trp medium for incorporation of tyrosine analogs contained 0.3 mM Tyr, and InvSC1 is prototrophic for the amino acid (see Materials and Methods for details). Unless Tyr is excluded from the cells, we suppose its intracellular concentration to rank in a comparable sub-millimolar range. The results in Figure S9 clearly show that Tyr is significantly activated at concentrations as low as 50 µM by the different AzRSs while the different ncAAs showed activation close to background at all tested concentrations (Figure 4, Figure 5, and Figure S9). Thus, from our results we would expect preferential activation of Tyr already at the normal concentration in the SC –Ura –Trp medium even in the presence of excess ncAA.

It might be possible that Tyr is not or only inefficiently charged onto the tRNA_CUA_ although it is efficiently activated by an o-aaRS. However, charging of tRNA_CUA_ with Tyr or the ncAAs by an o-aaRS, such as AzRS, PxRS, or BpaRS, has not been systematically analyzed so far. Most probably, this owes to the fact that only a couple of radiolabeled ncAAs, that are required for the classical aminoacylation assay, are commercially available. An alternative aminoacylation assay that involves radioactive tRNA rather than labeled amino acids has recently been described [44] and could be used for a future, more detailed analysis of the aminoacylation reaction of tRNA_CUA_ with ncAAs by the o-aaRSs.

Most strikingly, Bpa was incorporated into hSOD1 (Figure 2C) although the analog was not activated by BpaRS *in vitro* (Figure 5) when used at 5 mM. Due to the low solubility of Bpa in aqueous solution it was not possible to use higher concentrations in the activation assay. It is generally accepted that an amino acid must be activated before it can be charged onto a tRNA [2], [61]. *In vivo* activation and subsequent charging onto suppressor tRNA_CUA_ by an unspecific BpaRS can plausibly occur if Bpa is efficiently taken up into the cells and accumulates intracellularly to levels above 5 mM. This is possible if the substance is actively imported into the cells and piles up because it is not metabolized. Indeed, Wang and co-workers observed roughly 9 mM intracellular dansylalanine upon esterification of the amino acid [62]. Giese *et al.* recently demonstrated that fluorinated tryptophan is actively taken up into mammalian cells to an intracellular concentration exceeding the extracelluar by 70-fold [63]. A comparable accumulation could elevate the intracellular Bpa concentration above 5 mM when the cells are supplemented with 1 mM in the medium, as in our experiments. The assessment of the intracellular Bpa concentration would help to clarify this issue. In any case, the intracellular availability of the non-canonical amino acid is an important factor that governs the specificity of the o-aaRS and, thus, the fidelity of the incorporation [29].

In numerous studies, the site-specific incorporation of ncAAs into target proteins in response to amber codons was shown to occur with high fidelity and without significant amounts of canonical amino acids at the designated positions. However, reports on the catalytic properties of the evolved o-aaRSs as well as the intracellular availability and fate of the amino acid analogs are still rare exceptions 16,17,62. If the modification of proteins by ncAAs is to extend beyond the proof-of-principle level, thorough characterization of the incorporation systems is urgently required. Polysubstrate specificity, i.e. broad substrate tolerance of aaRSs appears to be a general phenomenon. Naturally occurring enzymes usually show it, and this trait can be exploited for the global incorporation of amino acid analogs into proteins [64]. O-aaRSs evolved to recognize a specific ncAA can also tolerate alternative substrates [52], [53]. This property could ease a broader applicability of the already existing o-pairs. However, natural enzymes and their cognate tRNAs are superior to the currently available orthogonal aaRS/tRNA_CUA_ pairs in terms of efficiency. In our opinion, the future strategies for the improvement of the o-pairs will enormously profit from the thorough characterization of the overall analog incorporation process. This involves the uptake of the ncAA into the cells, its cellular availability, the activation, and charging onto tRNA_CUA_ and finally the sequence context for the incorporation at the position of the amber stop codon [29], [65], [66]. The results of this thorough analysis will aid the design of o-pairs that can stand the comparison with the naturally occurring systems.

## Materials and Methods

### Materials

Unless otherwise indicated all chemicals were from Fluka (Neu-Ulm, Germany) or Merck (Darmstadt, Germany). Restriction endonucleases and T4 ligase were from New England Biolabs (Beverly, MA). ExTaq and rTaq DNA polymerases for proof-reading and standard PCR reactions, respectively, were from TaKaRa Bio Inc. (Saint-Germain-en-Laye, France), *PfuUltra* II HS-DNA-Polymerase for site-directed mutagenesis PCR was from Stratagene (La Jolla, CA).

### Construction of the tandem expression vectors for orthogonal pairs

The reconstruction of the expression vectors pTyr/tRNA_CUA_, pAz1/tRNA_CUA_ and pAz6/tRNA_CUA_ (Figure S1) were performed as described previously [18], [19], [27], [28]. Primers tRNA(CUA)template_fwd and tRNA(CUA)template_rev, containing the coding sequence for the *E. coli* tRNA_CUA_ gene, were annealed and the resulting double strand DNA was used for PCR amplification with primers tRNA5′ (contains an *Age*I cleavage site) and tRNA3′ (contains an *Nhe*I site, see Table S2 for primer sequences). The PCR product and the pESCTrp (Stratagene) target vector were both digested with *Nhe*I and *Age*I and ligated, which yielded ptRNA_CUA_. The *ADH1*-promoter for the expression of the *E. coli* TyrRS in *S. cerevisiae* was generated by PCR using the primers pADHf (*Age*I site) and pADHr (*Eco*RI site) together with genomic *S. cerevisiae* DNA as the template. The PCR product was digested with *Eco*RI and *Age*I. The *E. coli* TyrRS gene was PCR-amplified with the primers pESCTrp1 (*Eco*RI site) and pESCTrp2 (*Not*I site) and genomic *E. coli* DNA as the template. Afterwards the PCR product was digested with *Eco*RI and *Not*I. The pTyr/tRNA_CUA_ tandem vector was obtained by ligating *Age*I/*Not*I digested ptRNA_CUA_ with the *Age*I/*Eco*RI digested *ADH1*-promoter and *E. coli* TyrRS cut with *Eco*RI and *Not*I. The pAz1/tRNA_CUA_ descendant encoding the TyrRS mutant AzRS1 was generated by site directed mutagenesis PCR using the pTyr/tRNA_CUA_ vector as the template. The primer pair 5′-/3′Muta_N126N was used to introduce the silent mutation N126N. The additional mutations were introduced using the primer pairs 5′tyr_muta3306-1/pESCTrp2, 5′tyr_muta3306-2/pESCTrp2, and 5′tyr_muta3306-3/pESCTrp2. Each resulting mutant plasmid was used as the template for the subsequent mutagenesis PCR. AzRS6 was generated by introducing additional mutations into AzRS1 using mutagenesis primer pairs Thr37f/Thr37r, Ala183f/Ala183r, and Leu186f/Leu186r. Figure S2 shows the mutations that were introduced into TyrRS to generate the o-aaRSs. Mutagenesis primers are listed in Table S2. All vectors were verified by sequencing.

### Construction of the hSOD1(W33TAG) expression vector

The gene for the human superoxide dismutase (hSOD1) was PCR-amplified from the cDNA vector pOTB7 (ATCC number MGC-2325; LGC Promochem GmbH, Wesel, Germany) with the primers hSODfp (*Hind*III site) and hSODrp (*Eco*RI site). A hexahistidine-tag was introduced at the C-terminus of hSOD1 by primer hSODrp (see Table S2 for primer sequences). The PCR product and the high copy yeast-*E. coli* shuttle vector pYES2 (Invitrogen, Carlsbad, CA) were both digested with *Hind*III and *Eco*RI and ligated, yielding pYES2-hSOD1-6His. The tryptophan codon at position 33 of hSOD1 was mutated to an amber stop codon ATG by site directed mutagenesis PCR with the primers SODmutf and SODmutr (Table S2). Furthermore, the inducible *GAL* promoter on pYES2-hSOD1(W33TAG)-6His was exchanged for the stronger constitutive *PGK1* promoter via homologous recombination in yeast. To achieve this, the *GAL* promoter was excised from pYES2-hSOD1(W33TAG)-6His with *Spe*I and *Pvu*II. The DNA sequence of the *PGK1* promoter was amplified by PCR with primers PGK1fp and PGK1rp (Table S2) using genomic *S. cerevisiae* DNA as the template. In order to facilitate efficient homologous recombination, the primers PGK1fp and PGK1rp introduced flanking sequences of 43 nt and 35 nt, respectively to the *PGK1* promoter sequence. These sequences were homologous to the ends of the cut pYES2 vector. The *PGK1* PCR product with the flanking homology regions and the *Spe*I/*Pvu*II digested pYES2-hSOD1(W33TAG)-6His were co-transformed into *S. cerevisiae* strain InvSc1 (MATa/α his3Δ1/his3Δ1 leu2/leu2 trp1-289/trp1-289 ura3-52/ura3-52; Invitrogen) by the lithium acetate method [67] and clones carrying successfully recombined plasmids, which were designated pYES2-hSOD1(W33TAG)-6His, were selected on synthetic complete medium lacking uracil (SC –Ura; 1% glucose, 0.67% yeast nitrogen base (Difco Laboratories, MI), 1.92 g/L yeast synthetic drop-out medium supplement without uracil (Sigma, Deisenhofen, Germany)).

In a similar way, we exchanged the hexahistidine-tag for the *Strep*-tag II. The pYES2-hSOD1(W33TAG)-6His vector was linearized with *Eco*RI and the *Strep*-tag II coding sequence with appropriate homology hooks was amplified by PCR with primers hSOD1_Strep_fp and hSOD1_Strep_rp (see Table S2). Homologous recombination and screening of positive clones containing pYES2-hSOD1(W33TAG)-Strep was as described above for the promoter exchange.

### Expression and purification of hSOD1(W33TAG) variants containing Tyr analogs

The *S. cerevisiae* expression strain InvSc1 was co-transformed with one of the o-pair expression vectors and pYES2-hSOD1(W33TAG)-Strep by the lithium acetate method [67]. Positive transformants were selected on synthetic complete medium lacking uracil and tryptophan, but containing all other amino acids (SC –Ura –Trp; 1% glucose, 0.67% yeast nitrogen base (Difco), 1.4 g/L yeast synthetic drop-out medium supplement without histidine, leucine, tryptophan and uracil (Sigma), 76 mg/L histidine (Sigma), 380 mg/L leucine (Sigma); the medium contains 76 mg/L or 0.3 mM tyrosine disodium salt). For incorporation experiments, 5 mL SC –Ura –Trp medium were inoculated with the transformed expression strain and this starter culture was grown with vigorous shaking at 30°C over night. Subsequently, 50 mL SC –Ura –Trp were inoculated to an OD_600_ of 0.2 with the starter culture and incubated with shaking at 30°C for 24 h. Finally, we inoculated 500 mL SC –Ura –Trp medium supplemented with 1 mM of Tyr or one of the analogs with the pre-culture to an OD_600_ of 0.2. The amino acid was directly dissolved in the expression medium, which was then filter sterilized before inoculation. The hSOD1 variants were expressed with vigorous shaking at room temperature for 48 h. Cells were harvested by low speed centrifugation (3000×g, room temperature, 5 min) and the cell pellets were stored at −80°C until protein preparation.

To purify the hSOD1 variants, we lysed the yeast cells with Y-PER (Pierce, Rockford, IL) following the manufacturer's instructions. The lysate was cleared by centrifugation (14000×g, room temperature, 10 min) and applied onto a *Strep*-Tactin-column (1 mL column volume (CV); IBA BioTAGnology, Gottingen, Germany). After protein binding, the column was washed with 5 CVs of washing buffer (100 mM Tris/Cl pH 8.0, 150 mM NaCl, 1 mM EDTA). Bound protein was eluated 6 times with 0.5 CVs of elution buffer (100 mM Tris/Cl pH 8.0, 150 mM NaCl, 1 mM EDTA, 1 mM desthiobiotin) each. Eluates were pooled and concentrated to a total volume of 150 µl by ultrafiltration (Centricon YM-10, MWCO 10,000; Amicon, Beverly, MA).

### Immunoblotting

Of each concentrated hSOD1 variant, 20 µl were run on a 12% Laemmli gel [68]. After electrophoresis, the proteins were blotted onto nitrocellulose membrane (Schleicher and Schuell GmbH, Dassel, Germany) for immunodetection [69]. The membranes were blocked with TTBS (50 mM Tris/Cl, pH 8.0, 150 mM NaCl, 0.1% (v/v) Tween20) containing 1.5% (w/v) bovine serum albumin (SERVA Electrophoresis GmbH, Heidelberg, Germany) and probed with monoclonal mouse anti-*Strep*-tag II antibody MAB-Class (IBA BioTAGnology) as the first and horseradish peroxidase conjugated goat anti-mouse IgG (IBA BioTAGnology) as the second antibody. Tagged proteins were visualized by chemiluminescence detection (Pierce, Rockford, IL).

### Electro spray ionization mass spectrometry (ESI-MS)

For ESI-MS, 20 µl aliquots of the purified hSOD1 variants or the o-aaRSs were pre-separated on a Waters RP C4 column (300 Å pore size; 3.5 µm particle size; 100×2.1 mm; Waters GmbH, Eschborn, Germany) by eluting with a gradient from 20 to 90% B in A within 20 min, where eluent A was 0.05% (v/v) TFA in water and eluent B was 0.05% (v/v) TFA in acetonitrile. A flow rate of 250 µl/min was used. The masses of the eluted fractions were analyzed on a MicroTOF ESI-MS (Bruker Daltonics, Bremen, Germany).

### Cloning and expression of wild type TyrRS and the o-aaRSs for the ATP-PPi exchange assay

#### Cloning

The sequences encoding wild type TyrRS and the o-aaRSs were PCR amplified with primers pESCTrp1 and pESCTrp2 (Table S2) from corresponding o-pair expression vectors as templates. The primers contain an *EcoR*I or *Not*I cleavage site, respectively, flanked by additional nucleotides at their 5′-ends for efficient restriction. The PCR fragments were digested with *EcoR*I and *Not*I (New England Biolabs) and inserted into pET28a (Merck KGaA, Darmstadt, Germany) cleaved with the same enzymes, so that the hexahistidine-tag of the pET28a vector was attached to the N-terminus of the aaRSs. The resulting expression plasmids pET28a-H6-aaRS were sequence-verified.

#### Protein expression

The pET28a-H6-aaRS expression vectors were introduced into the *E. coli* strain B834 (DE3) (F^−^ *ompT hsdS*_B_(r_B_^−^ m_B_^−^) *gal dcm met* (DE3); Novagen Merck Chemicals, Nottingham, UK) by electroporation following standard laboratory procedures. Plasmid-harboring clones were selected and propagated in media containing 50 mg/L kanamycin. The sequences of the expression plasmids were verified by sequencing. For aaRS expression, the cells were grown in 1 liter LB (Luria Broth) medium at 37°C until they reached mid-log phase (OD_600_ 0.6–0.8). Then, gene expression was induced by the addition of 1 mM isopropyl-β-D-1-thiogalactopyranoside (IPTG; Applichem, Darmstadt, Germany) and was performed for 4–5 h at 25°C with vigorous shaking.

#### Protein purification

The cells were harvested by low speed centrifugation (3,200× g, 4°C, 20 min) and the cell pellet was resuspended in sodium phosphate buffer (50 mM NaH_2_PO_4_, 300 mM NaCl, pH 8.0) with 10 mM imidazole. After addition of 1 mg/mL DNase (Roche, Mannheim, Germany), RNase (Sigma), and lysozyme (Sigma) each, cells were ruptured by sonication and the homogenate cleared from cell debris by high speed centrifugation (30,000× g, 4°C, 30 min). The clear lysate was loaded onto a 5 mL HiTrap Chelating HP column (GE Healthcare, Munich, Germany), which was then washed with 10 CVs of sodium phosphate buffer containing 20 mM imidazole. Bound proteins were eluted by applying an imidazole gradient (20–250 mM) in sodium phosphate buffer. The elution fractions were analyzed by SDS-PAGE and those enriched in the desired aaRS were pooled and first dialyzed against 20 mM Tris·HCl pH 8.0 and then against aaRS-buffer (20 mM Tris/Cl pH 8.0, 150 mM KCl, 15 mM MgCl_2_ and 5 mM *β*-mercaptoethanol). The dialyzed fractions were concentrated by ultrafiltration (Vivaspin 20 MWCO 10,000; Sartorius AG, Göttingen, Germany). Finally, the concentration of the protein samples was determined by recording the absorption at 280 nm and purity was analyzed by SDS-Page (Figure S6) and ESI-MS mass analysis (Figure S7).

#### ATP-PPi exchange assay

The reaction mix contained 100 mM Tris/Cl pH 8.0, 80 mM MgCl_2_, 5 mM KF, 700 mM *β*-mercaptoethanol, 5.5 mM ATP, 0.1 mg/mL BSA, 2.2 mM [^32^P]-PPi (0.2 cpm/pmol; PerkinElmer, Rodgau, Germany), 5 µM of the o-aaRS (or 1 µM of wild type TyrRS), and 5 mM (or less, as indicated) of Tyr or Tyr analogs in a final volume of 200 µl. For the assay, 20 µM stock solutions of the purified aaRSs in aaRS-buffer were used. All amino acids were dissolved in 0.01 M HCl at a concentration of 12.5 mM and 80 µl of these amino acid solutions were used in each reaction mix (200 µl) to obtain 5 mM of amino acid final concentration. Lower amino acid concentrations were prepared by dilution in 0.01 M HCl, and 0.01 M HCl was used as the negative control (w/o aa). Each value was determined twice. After 15 min at 37°C, the reaction was quenched by mixing 100 µl of the reaction mix with 600 µl of 240 mM sodium pyrophosphate solution containing 70% (v/v) perchloric acid. [^32^P]-ATP formation was followed by specific absorption to 200 µl of 7.5% (w/v) activated charcoal. The suspension was thoroughly mixed and filtered through Whatman GF/F paper (Whatman International Ltd, Maidstone Kent, UK). Filters were washed twice with 10 mL of water before immersion in scintillation solution (Rotiszint eco plus; Carl Roth GmbH Co., Karlsruhe, Germany) to determine the amount of adsorbed radioactivity.

## Supporting Information

Figure S1 **Plasmid map of the tandem expression vector for the orthogonal aaRS/tRNA_CUA_ pairs.** The aaRS is expressed under the strong, constitutive *ADH1* promoter on a yeast/*E. coli* shuttle vector containing an ampicillin resistance gene (*AmpR*) and the ColE1 origin of replication for selection and propagation in *E. coli*, respectively. The *TRP1* auxotrophy marker and the 2µ origin of replication ensure plasmid maintenance in yeast. The amber suppressor tRNA (tRNA_CUA_) expression cassettes are detailed in Table 1. The following tandem expression vectors were used in this work (the original o-aaRS nomenclature is given in brackets): pTyr/tRNA_CUA_ for TyrRS (TyrRS [18]), pAz1/tRNA_CUA_ for AzRS1 (*p*-azidoPheRS1 [18]), pAz3/3SUP-tRNA_CUA_ for AzRS3 (*p*-azidoPheRS3 [29]), pAz6/tRNA_CUA_ for AzRS6 (*p*-azidoPheRS6 [18]), pPR1/3SUP-tRNA_CUA_ for PxRS1 (*p*-PpaRS1 [29]) and pBpa/tRNA_CUA_ for BpaRS (*p*-benzoylPheRS2 [18]).
(TIF)

Figure S2 **DNA and protein sequence alignment of TyrRS and the o-aaRSs.** Mutated bases and exchanged amino acid residues are highlighted in color.
(TIF)

Figure S3 **The hSOD1(W33TAG) expression vector.** Constitutive expression of hSOD1 with an in-frame amber stop codon (TAG) in position 33 and a C-terminal *Strep*-tag II is driven by the constitutive *PGK1* promoter. The yeast/*E. coli* shuttle vector contains an ampicillin resistance gene (*AmpR*) and the ColE1 origin of replication for selection and propagation in *E. coli*, respectively. The *URA3* auxotrophy marker and the 2µ origin of replication ensure plasmid maintenance in yeast.
(TIF)

Figure S4 **DNA and protein sequence of the hSOD1(W33TAG) open reading frame with a C-terminal** ***Strep*** **-tag II.** The position of the amber stop codon (bold boxed) is indicated as X (bold black) in the protein sequence, hSOD1 is highlighted in green and the *Strep*-tag II in orange.
(TIF)

Figure S5 **Intracellular expression of the o-aaRSs from different orthogonal pairs.** Sample preparation and immunodetection are described in Methods S1. For immunodetection, a C-terminal hexahistidine-tag was added to AzRS1 on pAz1/tRNA_CUA_ by homologous recombination as described in Methods S1. AzRS3 and PxRS1 on pAz3/3SUP-tRNA_CUA_ and pPR1/3SUP-tRNA_CUA_, respectively, originally contained a C-terminal hexahistidine-tag [29]. While only a single clone was analyzed for AzRS1(His) expression (lane 1), three different clones each were analyzed for expression of AzRS3 (lanes 2–4), and PxRS1 (lanes 5–7). The calculated molecular weight of the o-aaRSs is 48 kDa. M, molecular weight marker; +, wild type hSOD1 with a C-terminal hexahistidine-tag (positive control); -, empty lane.
(TIF)

Figure S6 **Purified** ***E. coli*** **wild type TyrRS and o-aaRSs.** aaRSs were expressed in *E. coli* and purified by Ni-NTA affinity chromatography. The proteins were concentrated by ultrafiltration and analyzed by SDS-PAGE. The calculated molecular weight of TyrRS and the o-aaRSs is 51 kDa. 1, TyrRS; 2, AzRS1; 3, AzRS6; 4, AzRS3; 5, BpaRS; M, molecular weight marker. The gel was cut between lanes 4 and 5 to remove irrelevant lanes.
(TIF)

Figure S7 **ESI-MS analysis of** ***E. coli*** **wild type TyrRS and the o-aaRSs shown in Figure S6 and Table S1.** TyrRS (A); AzRS1 (B); AzRS3 (C); AzRS6 (D); BpaRS (E).
(TIF)

Figure S8 **ATP-PPi exchange assay of TyrRS with different concentrations of Tyr.** Different concentrations of Tyr were used to determine the substrate range for non-saturated enzyme activity (A) and for substrate saturation (B). In the negative control, no amino acid was added to the reaction mix (w/o aa). TyrRS was used at a concentration of 1 µM. The data in (A) and (B) were collected in one series of experiments each and the ATP formation with each tyrosin concentration was determined in duplicate. Mean values are shown; the bars denote the discrete values.
(TIF)

Figure S9 **Activation of Tyr, AzF and AmF at low concentrations by the different AzRSs.** In order to exclude effects of substrate inhibition in the ATP-PPi assay, 5 µM each of AzRS1 (A), AzRS3 (B), and AzRS6 (C) were incubated with Tyr, AzF, and AmF at concentrations that had caused linear activation of Tyr by TyrRS (refer to Figure S8). In the negative control, no amino acid was added to the reaction mix (w/o aa). The data for each aaRS were all collected in one series of experiments. Mean values of duplicates are shown; the bars denote the discrete values.
(TIF)

Figure S10 **ESI mass spectrum of the PxF preparation.** Among other unidentified impurities, approximately 1% Tyr (corresponding mass indicated in red in the lower panel) was present, most probably originating from the chemical synthesis [19]. The lower panel shows the same mass spectrum as the upper panel albeit at a magnified intensity scale (×10^6^ in the upper *vs.* ×10^4^ in the lower panel).
(TIF)

Figure S11 **Activation of different amino acids and their analogs by** ***E. coli*** **PheRS (Gly294Phe).** The negative control did not contain an amino acid (w/o aa). PheRS (Gly294Phe) [56] was added at a concentration of 3 µM and the amino acid analogs at 5 mM. Besides the amino acids shown in Figure 1, the following ncAAs were used: MY (O-methyl-L-tyrosine), IF (*p*-iodo-L-phenylalanine), FA (L-(2-furyl)-alanine), AA (azulenyl-L-alanine), 6ClW (6-chloro-DL-tryptophan), 5BrW (5-bromo-DL-tryptophan), BT (benzothienyl-L-alanine), Nal (naphthyl-L-alanine). The data were all collected in one series of experiments. Mean values of duplicates are shown; the bars denote the discrete values.
(TIF)

Table S1 **Mass analysis of** ***E. coli*** **wild type TyrRS and the different o-aaRSs.**
(DOC)

Table S2 **DNA primer sequences.**
(DOC)

Methods S1 **Contains the technical details for the analysis of the intracellular expression of the TyrRS mutants.**
(DOC)
