# Supplementary figures and images for: Performance Analysis of Orthogonal Pairs Designed for an Expanded Eukaryotic Genetic Code
Source: PLoS One. 2012 Apr 6;7(4):e31992. doi: 10.1371/journal.pone.0031992 (PMC3320878; doi:10.1371/journal.pone.0031992)

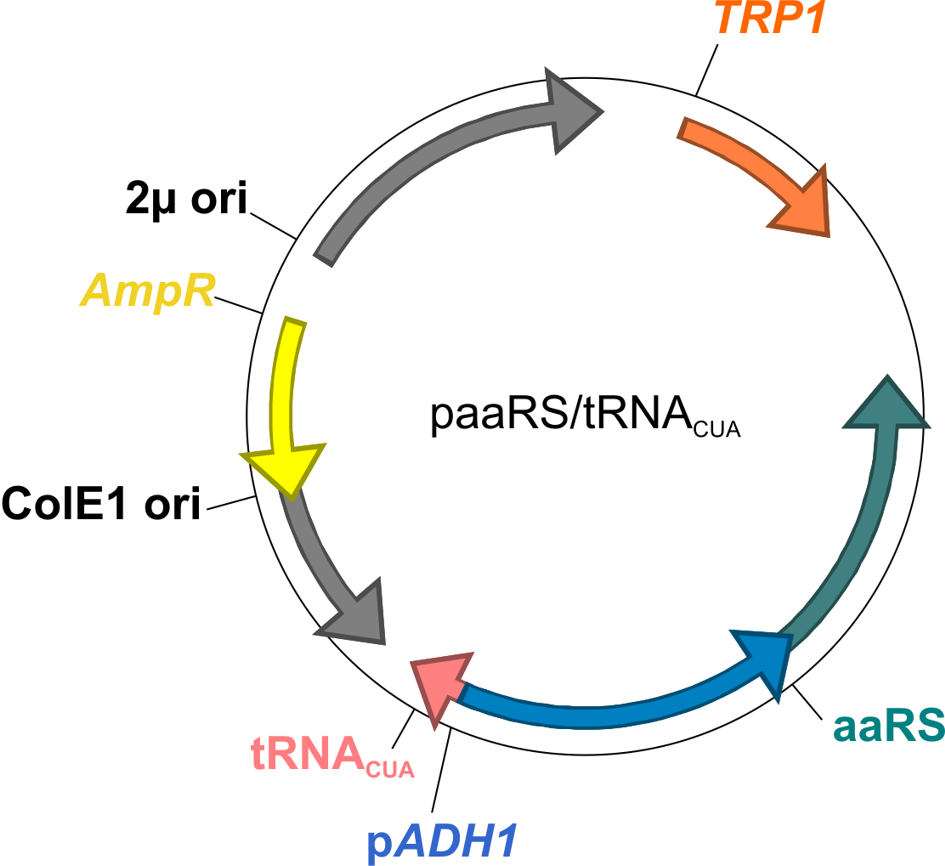

Supplement: Figure S1 — Plasmid map of the tandem expression vector for the orthogonal aaRS/tRNACUA pairs. The aaRS is expressed under the strong, constitutive ADH1 promoter on a yeast/E. coli shuttle vector containing an ampicillin resistance gene (AmpR) and the ColE1 origin of replication for selection and propagation in E. coli, respectively. The TRP1 auxotrophy marker and the 2µ origin of replication ensure plasmid maintenance in yeast. The amber suppressor tRNA (tRNACUA) expression cassettes are detailed in Table 1. The following tandem expression vectors were used in this work (the original o-aaRS nomenclature is given in brackets): pTyr/tRNACUA for TyrRS (TyrRS [18]), pAz1/tRNACUA for AzRS1 (p-azidoPheRS1 [18]), pAz3/3SUP-tRNACUA for AzRS3 (p-azidoPheRS3 [29]), pAz6/tRNACUA for AzRS6 (p-azidoPheRS6 [18]), pPR1/3SUP-tRNACUA for PxRS1 (p-PpaRS1 [29]) and pBpa/tRNACUA for BpaRS (p-benzoylPheRS2 [18]). (TIF) [file pone.0031992.s001.tif]

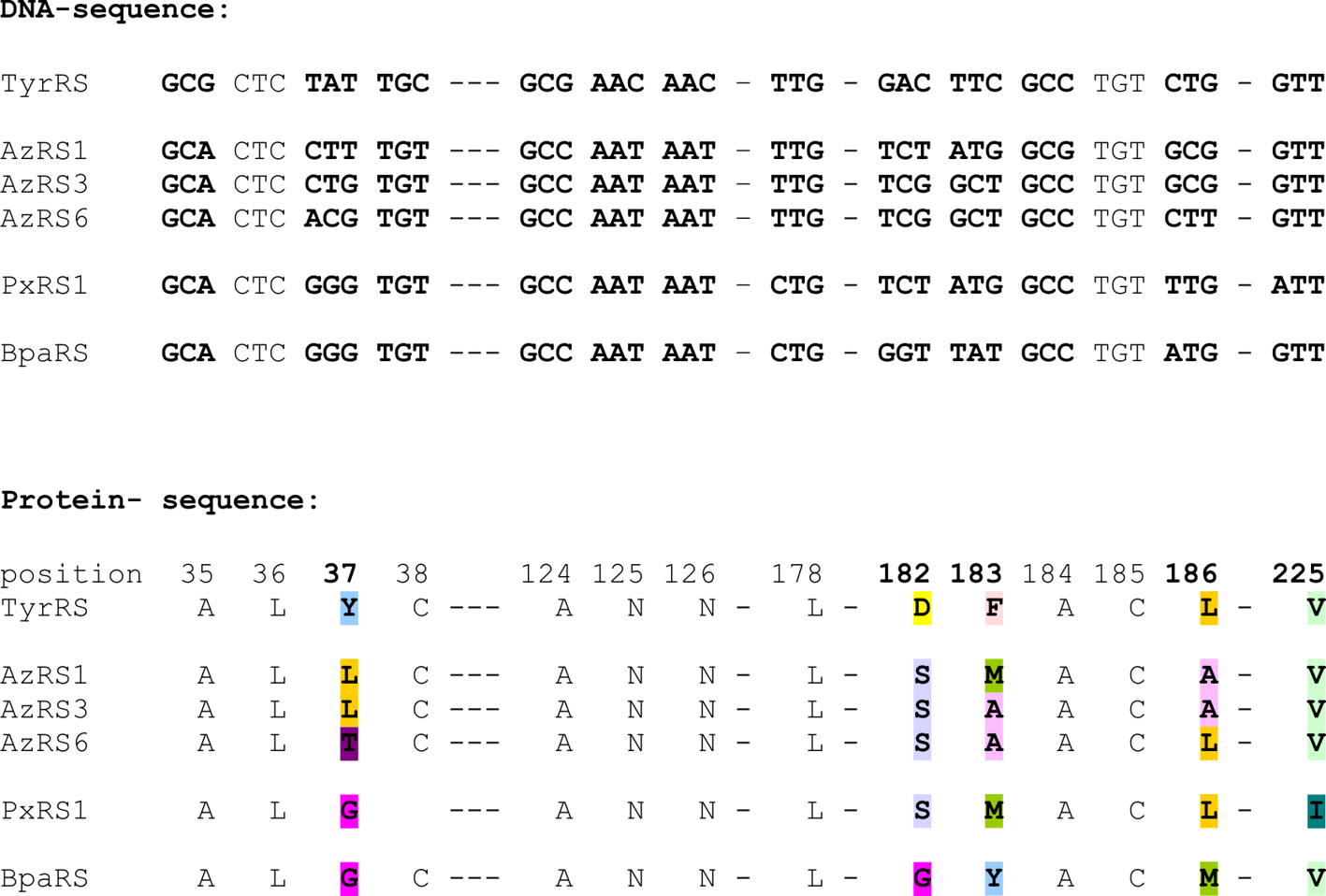

Supplement: Figure S2 — DNA and protein sequence alignment of TyrRS and the o-aaRSs. Mutated bases and exchanged amino acid residues are highlighted in color. (TIF) [file pone.0031992.s002.tif]

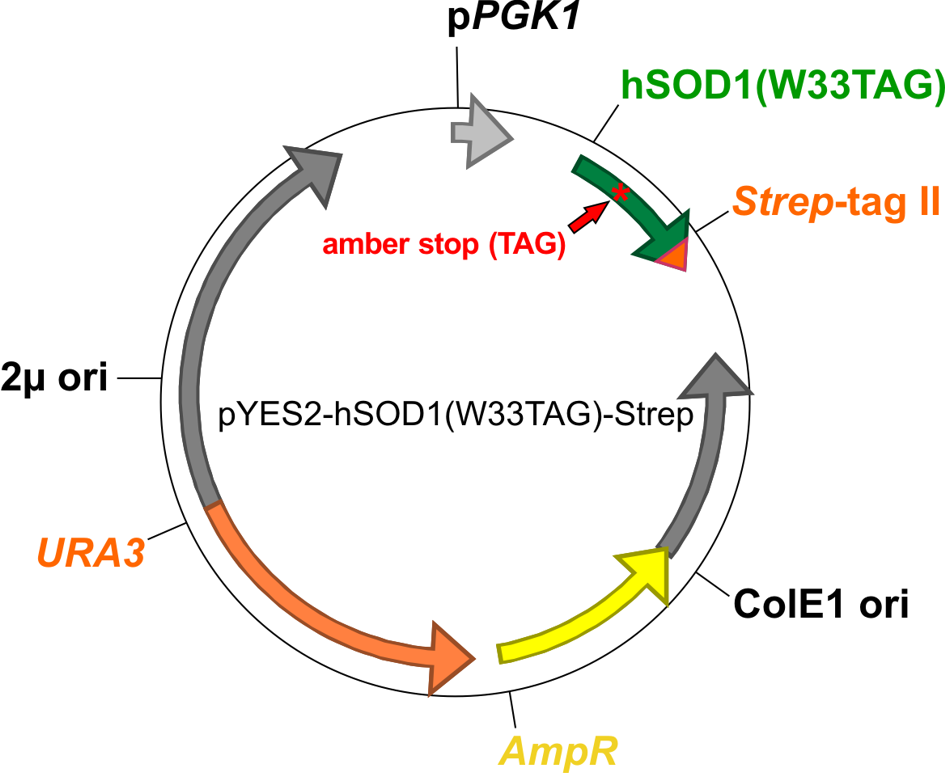

Supplement: Figure S3 — The hSOD1(W33TAG) expression vector. Constitutive expression of hSOD1 with an in-frame amber stop codon (TAG) in position 33 and a C-terminal Strep-tag II is driven by the constitutive PGK1 promoter. The yeast/E. coli shuttle vector contains an ampicillin resistance gene (AmpR) and the ColE1 origin of replication for selection and propagation in E. coli, respectively. The URA3 auxotrophy marker and the 2µ origin of replication ensure plasmid maintenance in yeast. (TIF) [file pone.0031992.s003.tif]

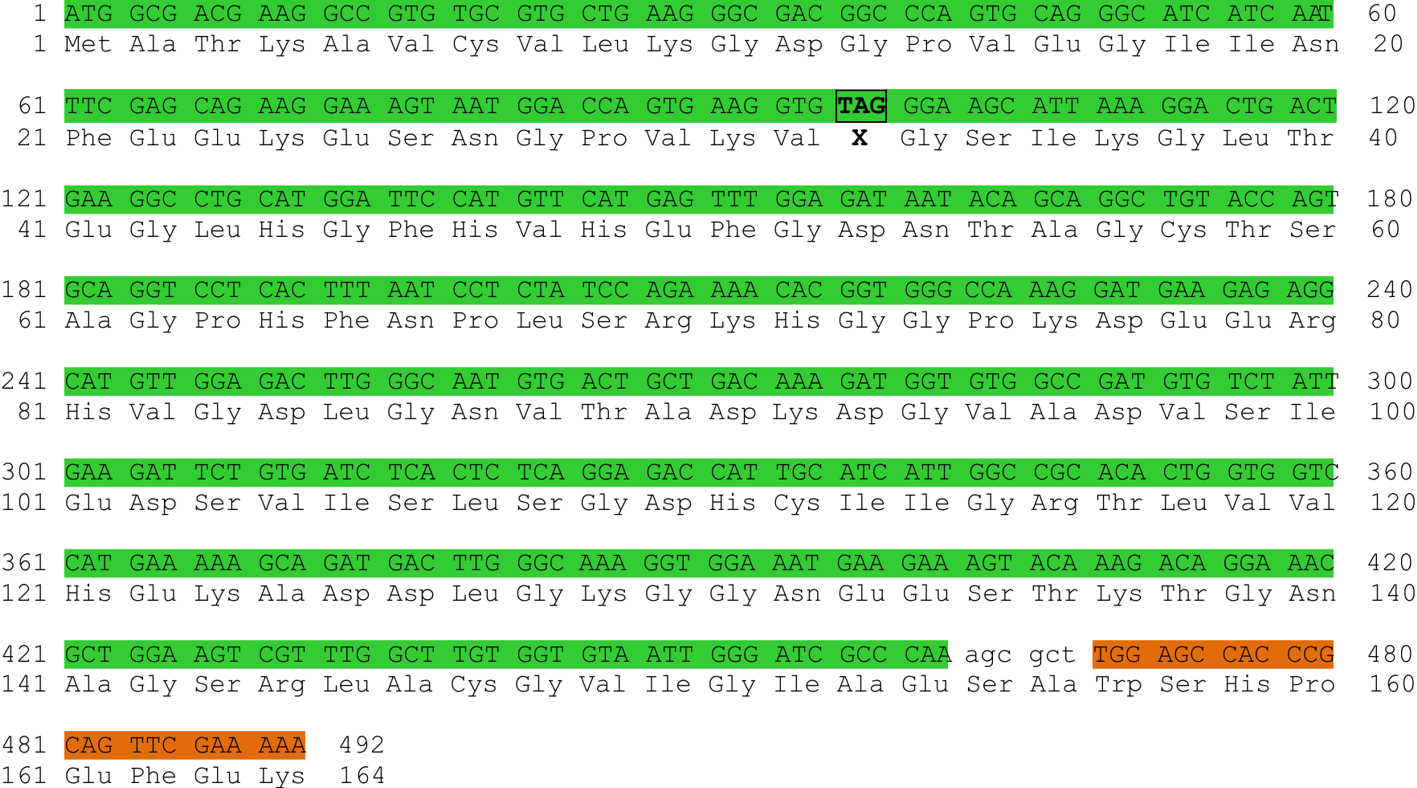

Supplement: Figure S4 — DNA and protein sequence of the hSOD1(W33TAG) open reading frame with a C-terminal Strep -tag II. The position of the amber stop codon (bold boxed) is indicated as X (bold black) in the protein sequence, hSOD1 is highlighted in green and the Strep-tag II in orange. (TIF) [file pone.0031992.s004.tif]

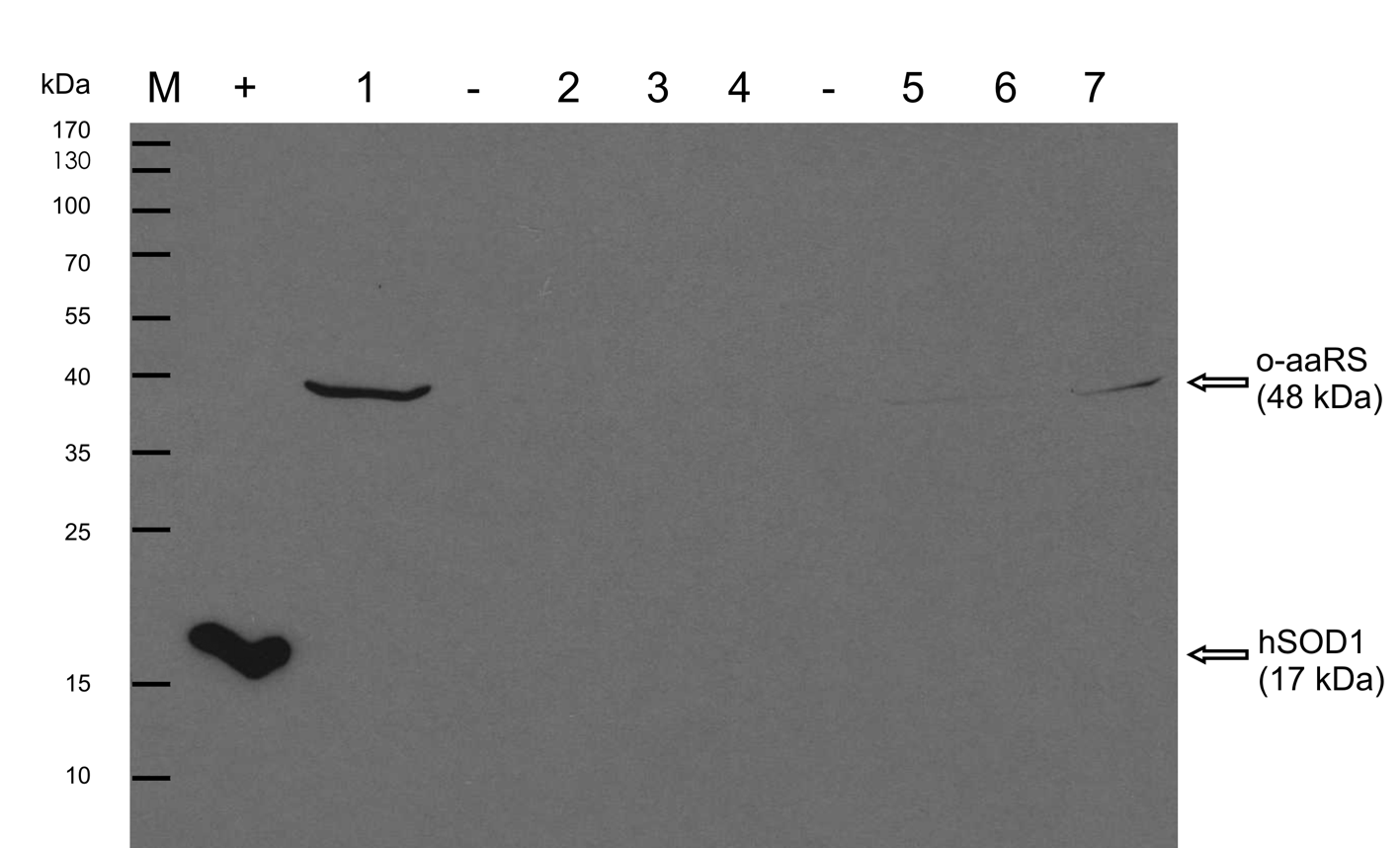

Supplement: Figure S5 — Intracellular expression of the o-aaRSs from different orthogonal pairs. Sample preparation and immunodetection are described in Methods S1. For immunodetection, a C-terminal hexahistidine-tag was added to AzRS1 on pAz1/tRNACUA by homologous recombination as described in Methods S1. AzRS3 and PxRS1 on pAz3/3SUP-tRNACUA and pPR1/3SUP-tRNACUA, respectively, originally contained a C-terminal hexahistidine-tag [29]. While only a single clone was analyzed for AzRS1(His) expression (lane 1), three different clones each were analyzed for expression of AzRS3 (lanes 2–4), and PxRS1 (lanes 5–7). The calculated molecular weight of the o-aaRSs is 48 kDa. M, molecular weight marker; +, wild type hSOD1 with a C-terminal hexahistidine-tag (positive control); -, empty lane. (TIF) [file pone.0031992.s005.tif]

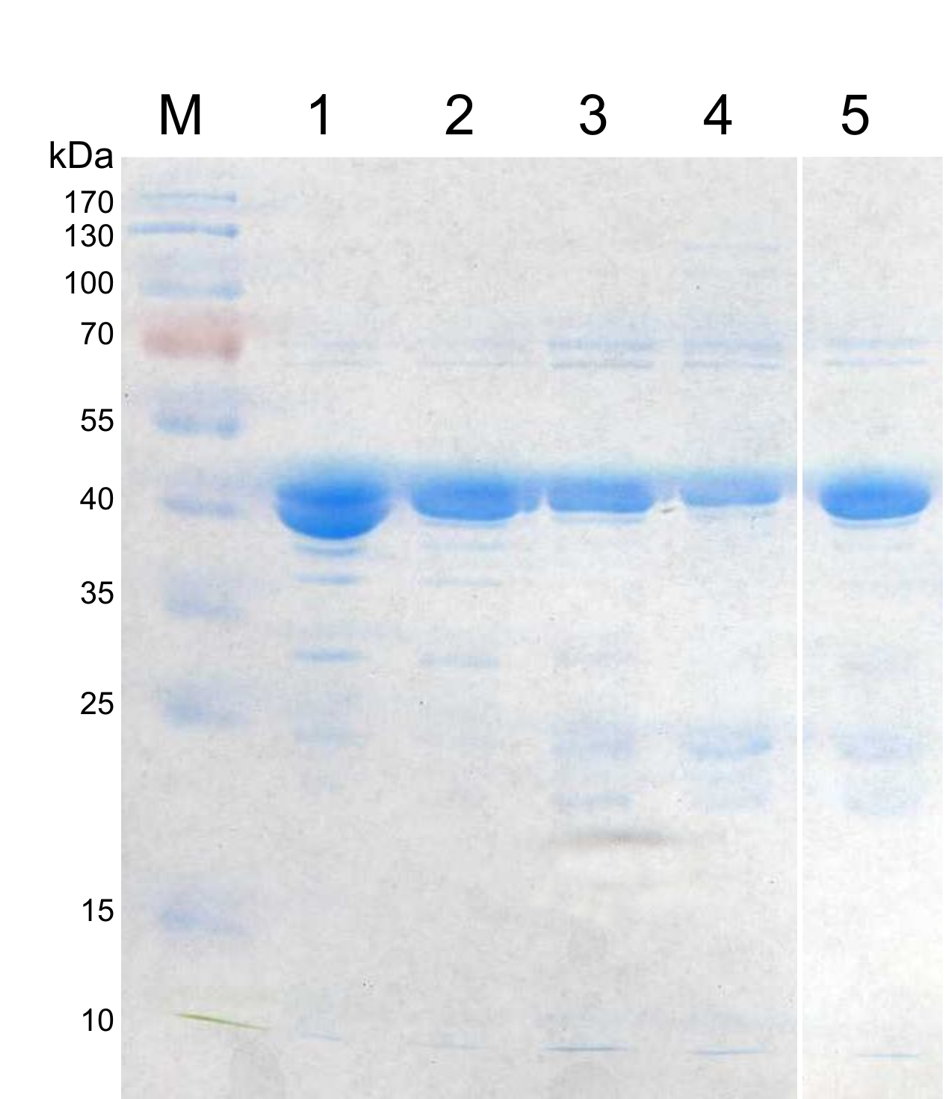

Supplement: Figure S6 — Purified E. coli wild type TyrRS and o-aaRSs. aaRSs were expressed in E. coli and purified by Ni-NTA affinity chromatography. The proteins were concentrated by ultrafiltration and analyzed by SDS-PAGE. The calculated molecular weight of TyrRS and the o-aaRSs is 51 kDa. 1, TyrRS; 2, AzRS1; 3, AzRS6; 4, AzRS3; 5, BpaRS; M, molecular weight marker. The gel was cut between lanes 4 and 5 to remove irrelevant lanes. (TIF) [file pone.0031992.s006.tif]

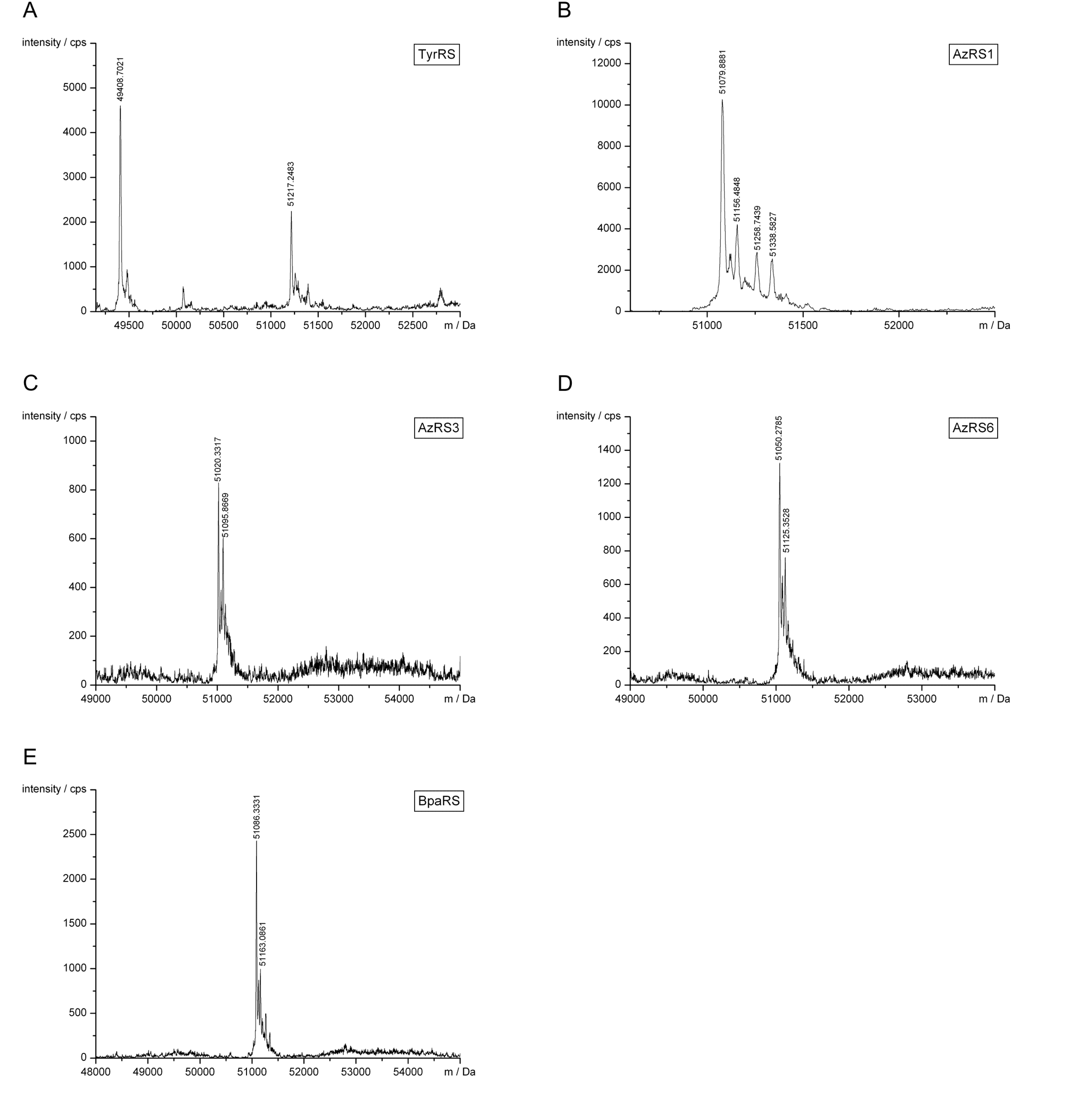

Supplement: Figure S7 — ESI-MS analysis of E. coli wild type TyrRS and the o-aaRSs shown in Figure S6 and Table S1. TyrRS (A); AzRS1 (B); AzRS3 (C); AzRS6 (D); BpaRS (E). (TIF) [file pone.0031992.s007.tif]

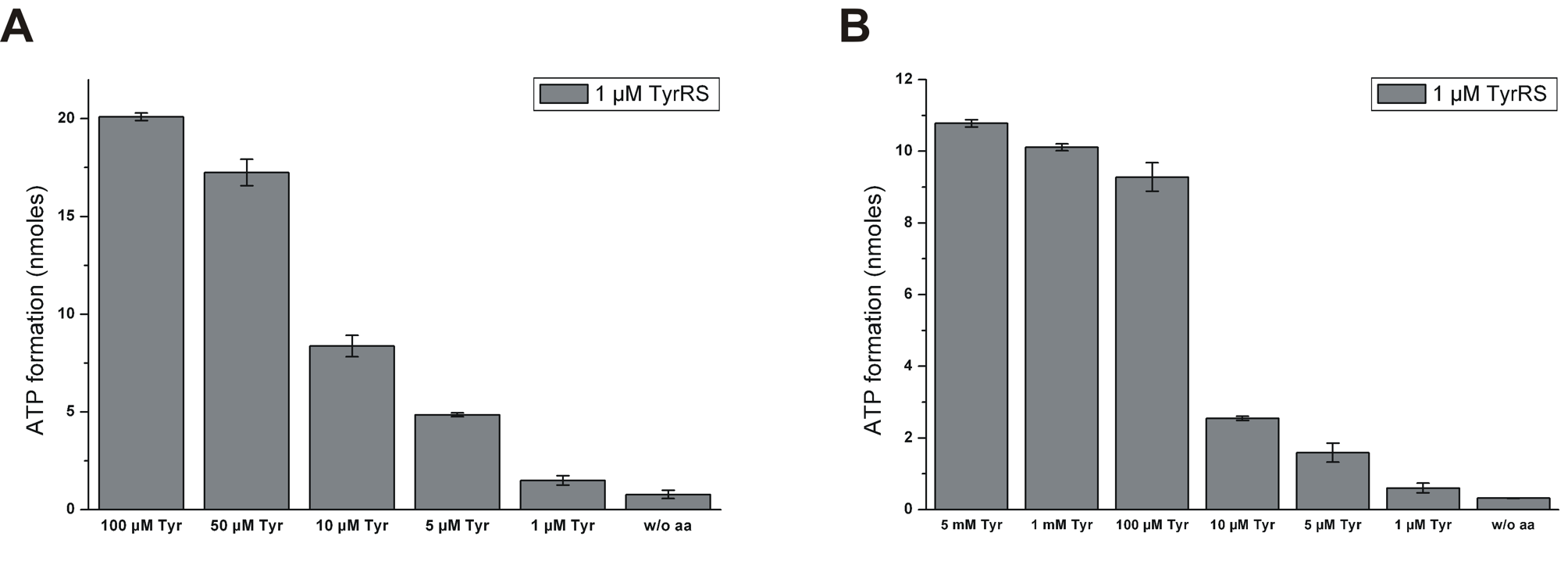

Supplement: Figure S8 — ATP-PPi exchange assay of TyrRS with different concentrations of Tyr. Different concentrations of Tyr were used to determine the substrate range for non-saturated enzyme activity (A) and for substrate saturation (B). In the negative control, no amino acid was added to the reaction mix (w/o aa). TyrRS was used at a concentration of 1 µM. The data in (A) and (B) were collected in one series of experiments each and the ATP formation with each tyrosin concentration was determined in duplicate. Mean values are shown; the bars denote the discrete values. (TIF) [file pone.0031992.s008.tif]

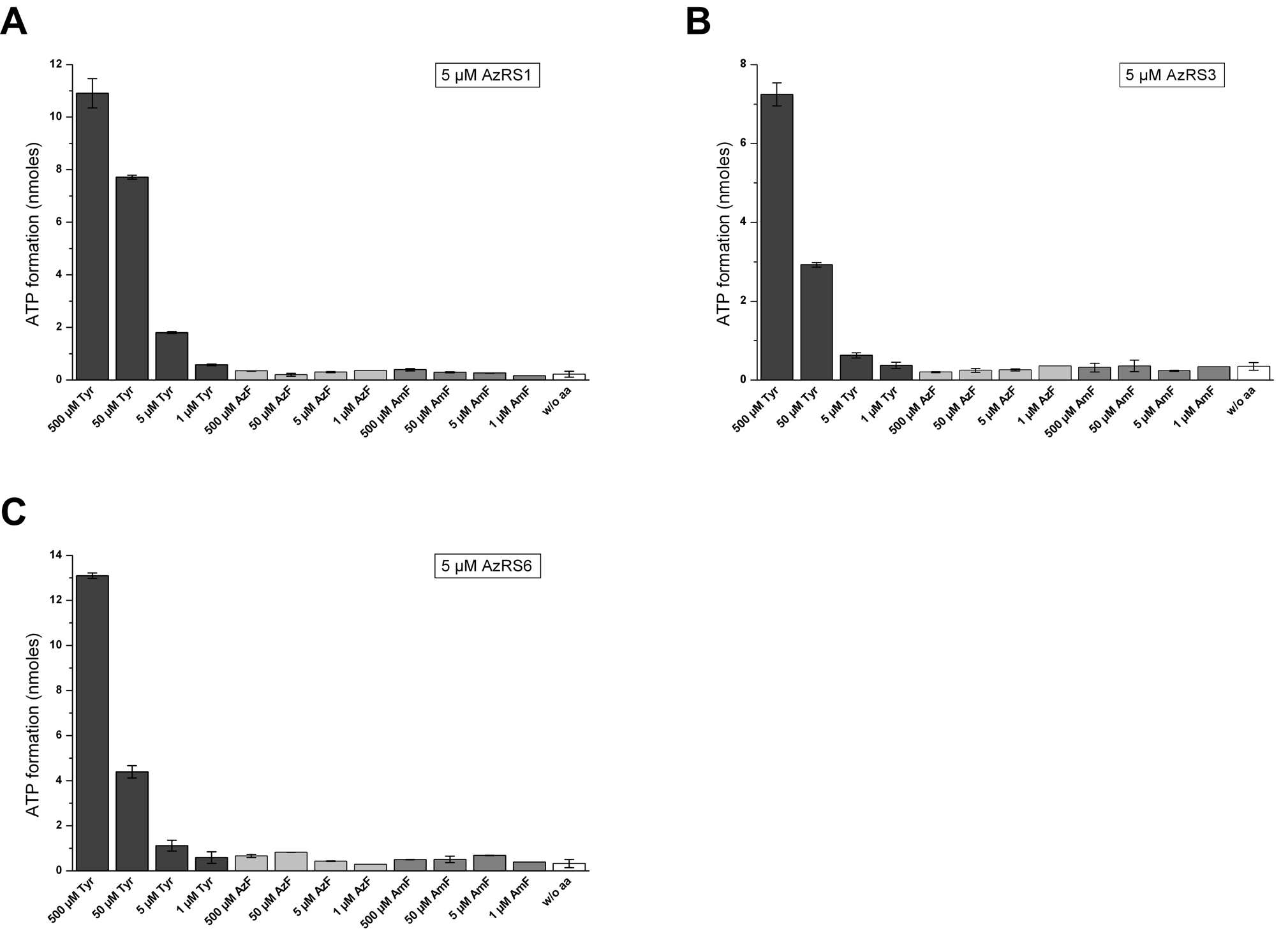

Supplement: Figure S9 — Activation of Tyr, AzF and AmF at low concentrations by the different AzRSs. In order to exclude effects of substrate inhibition in the ATP-PPi assay, 5 µM each of AzRS1 (A), AzRS3 (B), and AzRS6 (C) were incubated with Tyr, AzF, and AmF at concentrations that had caused linear activation of Tyr by TyrRS (refer to Figure S8). In the negative control, no amino acid was added to the reaction mix (w/o aa). The data for each aaRS were all collected in one series of experiments. Mean values of duplicates are shown; the bars denote the discrete values. (TIF) [file pone.0031992.s009.tif]

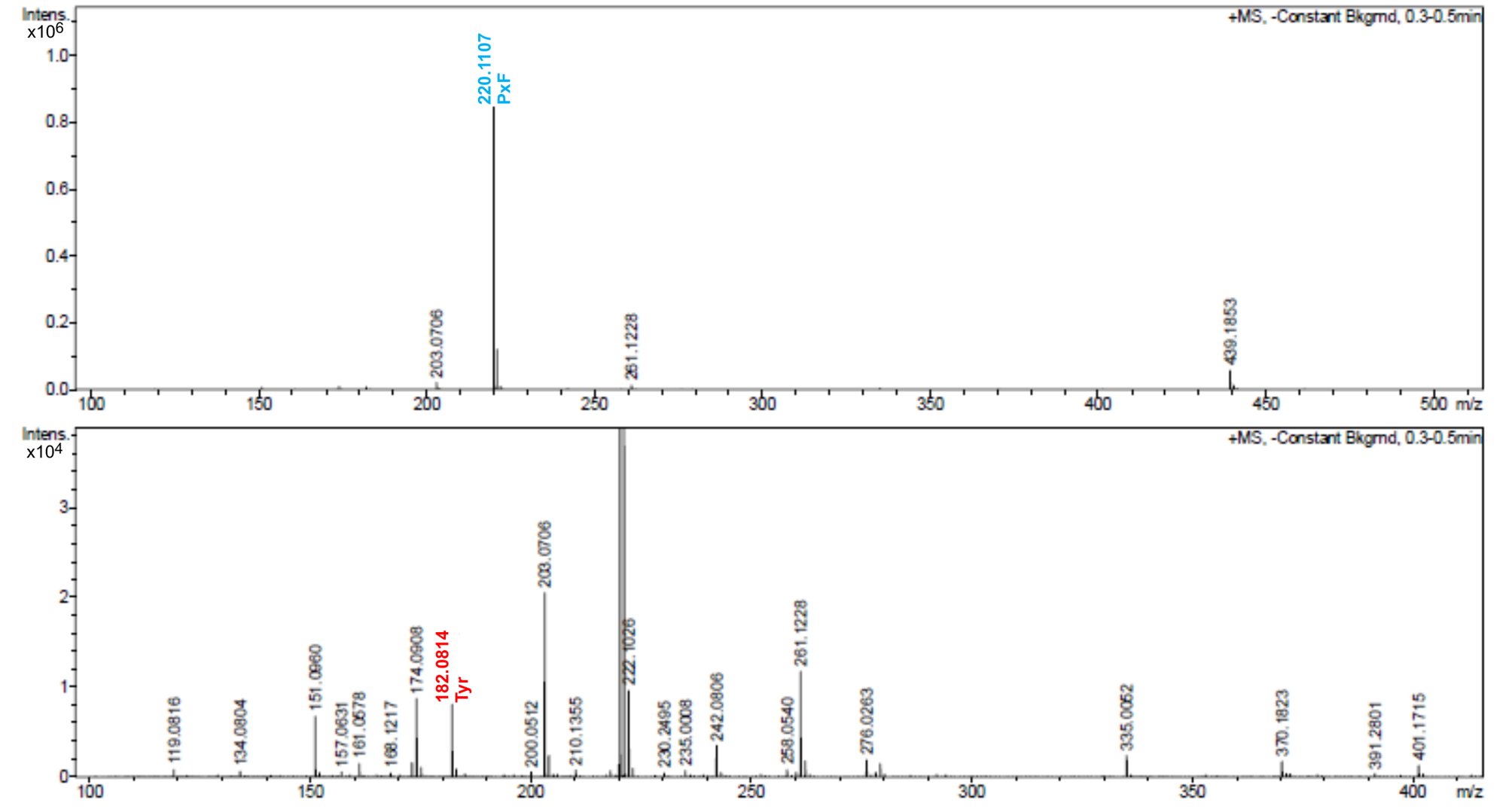

Supplement: Figure S10 — ESI mass spectrum of the PxF preparation. Among other unidentified impurities, approximately 1% Tyr (corresponding mass indicated in red in the lower panel) was present, most probably originating from the chemical synthesis [19]. The lower panel shows the same mass spectrum as the upper panel albeit at a magnified intensity scale (×106 in the upper vs. ×104 in the lower panel). (TIF) [file pone.0031992.s010.tif]

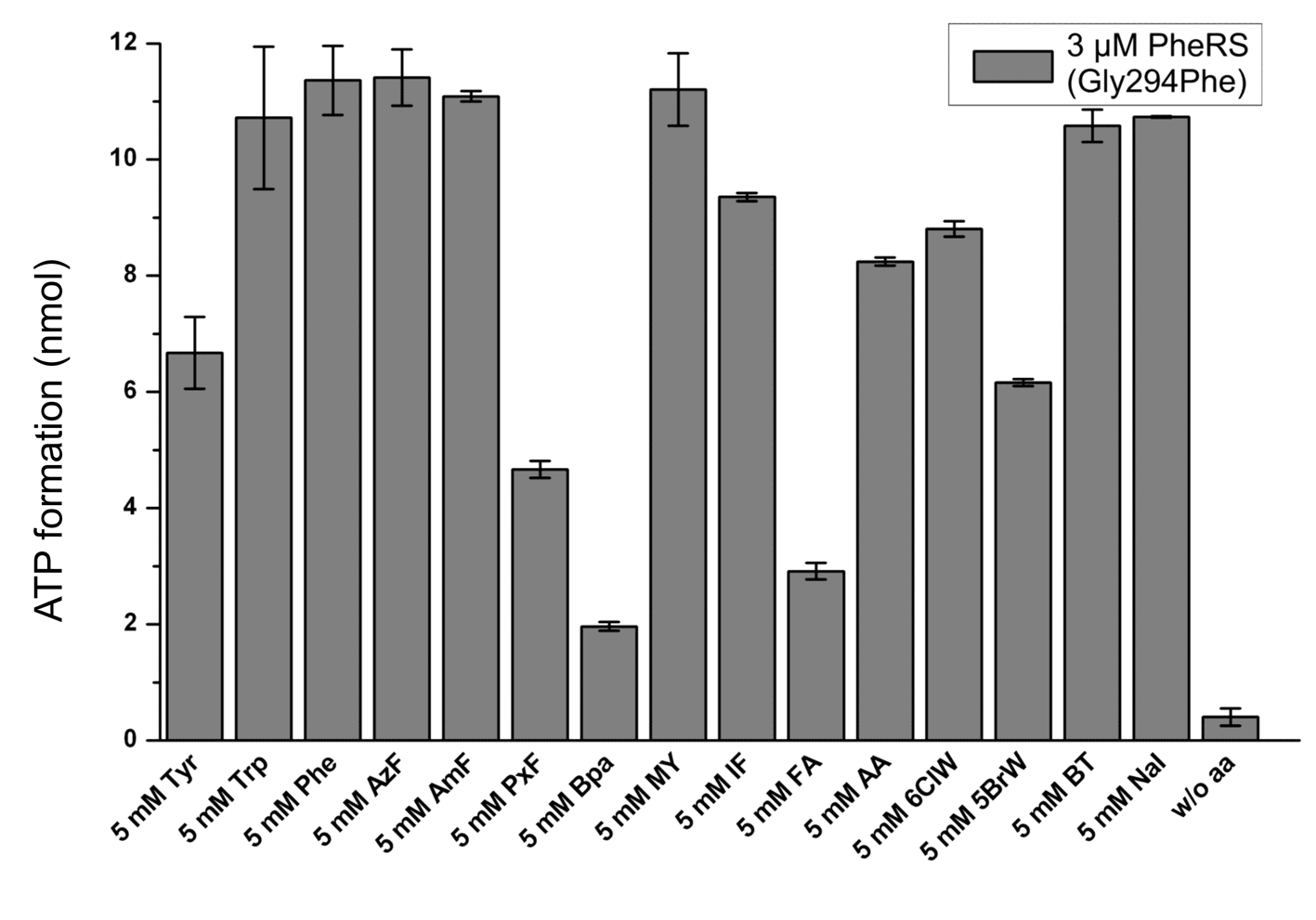

Supplement: Figure S11 — Activation of different amino acids and their analogs by E. coli PheRS (Gly294Phe). The negative control did not contain an amino acid (w/o aa). PheRS (Gly294Phe) [56] was added at a concentration of 3 µM and the amino acid analogs at 5 mM. Besides the amino acids shown in Figure 1, the following ncAAs were used: MY (O-methyl-L-tyrosine), IF (p-iodo-L-phenylalanine), FA (L-(2-furyl)-alanine), AA (azulenyl-L-alanine), 6ClW (6-chloro-DL-tryptophan), 5BrW (5-bromo-DL-tryptophan), BT (benzothienyl-L-alanine), Nal (naphthyl-L-alanine). The data were all collected in one series of experiments. Mean values of duplicates are shown; the bars denote the discrete values. (TIF) [file pone.0031992.s011.tif]
