# Supplementary material for: Performance Analysis of Orthogonal Pairs Designed for an Expanded Eukaryotic Genetic Code
Source: PLoS One. 2012 Apr 6;7(4):e31992. doi: 10.1371/journal.pone.0031992 (PMC3320878; doi:10.1371/journal.pone.0031992)
Supplement: Table S1 — Mass analysis of E. coli wild type TyrRS and the different o-aaRSs. (DOC) [file pone.0031992.s012.doc]

# Supporting Information

# Performance analysis of orthogonal pairs designed for an expanded eukaryotic genetic code

## Sebastian Nehring^1^, Nediljko Budisa^1^, Birgit Wiltschi^2,3,4^*

1 Department of Biocatalysis, Technical University of Berlin, Berlin, Germany

2 BIOSS - Centre for Biological Signalling Studies, Albert-Ludwigs-University Freiburg, Freiburg, Germany

2 Faculty of Biology, Albert-Ludwigs-University Freiburg, Freiburg, Germany

4 Present Address: Austrian Centre of Industrial Biotechnology, Graz, Austria

* To whom correspondence should be addressed.
E-mail: birgit.wiltschi@acib.at

Table S1 Mass analysis of *E. coli* wild type TyrRS and the different o-aaRSs

| Protein | Calculated mass (Da) | Detected mass (Da) | Δm (Da) |
| --- | --- | --- | --- |
| TyrRS | 51216.509 | 51217.248 | +0.739 |
| TyrRS (N-term. truncated) | 49408.616 | 49408.702 | +0.086 |
| AzRS1 | 51080.426 | 51079.888 | -0.538 |
| AzRS3 | 51020.307 | 51020.332 | +0.025 |
| AzRS6 | 51050.333 | 51050.279 | -0.055 |
| BpaRS | 51086.389 | 51086.333 | -0.056 |

All masses were calculated without N-terminal Met; Met was cleaved off because of Gly at position 2. Unexpectedly, a truncated TyrRS variant was found, in which the N-terminus had been cleaved off at the thrombin cleavage site (encoded on the pET28a expression vector) between the hexahistidine-tag and the TyrRS. Most probably, the truncated, untagged variant was co-purified by forming heterodimers with the full length enzyme. The N-terminally truncated TyrRS accounted for approximately 60% of the total TyrRS preparation (refer to the SDS gel in Figure S6, lane 1). Corresponding mass spectra are shown in Figure S7.
