# Supplementary material for: Performance Analysis of Orthogonal Pairs Designed for an Expanded Eukaryotic Genetic Code
Source: PLoS One. 2012 Apr 6;7(4):e31992. doi: 10.1371/journal.pone.0031992 (PMC3320878; doi:10.1371/journal.pone.0031992)
Supplement: Table S2 — DNA primer sequences. (DOC) [file pone.0031992.s013.doc]

# Supporting Information

# Performance analysis of orthogonal pairs designed for an expanded eukaryotic genetic code

## Sebastian Nehring^1^, Nediljko Budisa^1^, Birgit Wiltschi^2,3,4^*

1 Department of Biocatalysis, Technical University of Berlin, Berlin, Germany

2 BIOSS - Centre for Biological Signalling Studies, Albert-Ludwigs-University Freiburg, Freiburg, Germany

2 Faculty of Biology, Albert-Ludwigs-University Freiburg, Freiburg, Germany

4 Present Address: Austrian Centre of Industrial Biotechnology, Graz, Austria

* To whom correspondence should be addressed.
E-mail: birgit.wiltschi@acib.at

Table S2 DNA primer sequences

| Name | 5’ → 3’ Sequence | cleavage sites / tags |
| --- | --- | --- |
| tRNA(CUA)template_fwd | GGTGGGGTTCCCGAGCGGCCAAAGGGAGCAGACTCTAAATCTGCCGTCATCGACCTCGAAGGTTCGAATCCTTCCCCCACCAC | - |
| tRNA(CUA)template_rev | gtggtgggggaaggattcgaaccttcgaGgtcgatgacggcagattTAGagtctgctccctttggccgctcgggaaccccacc | - |
| tRNA5’ | GGGGGG***ACCGGT***AAGCTTCCCGATAAGGGAGCAGGCCAGTAAAAAGCATTACCCCGTGGTGGGTTCCCGA | *Age*I |
| tRNA3’ | GGCGGC***GCTAGC***GGGAAGTTCAGGGACTTTTGAAAAAAATGGTGGTGGGGGAAGGAT | *Nhe*I |
| pADHf | GGGGGG***ACCGGT***CGGGATCGAAGAAATGATGGTAAATGA AATAGGAAATCAAGG | *Age*I |
| pADHr | GGGGGG***GAATTC***AGTTGATTGTATGCTTGGTATAGCTTGAAATATTGTGCAGAAAAAGAAAC | *Eco*RI |
| pESCTrp1 | TCATAACGA***GAATTC***ATG GCAAGCAGTAACTTG | *Eco*RI |
| pESCTrp2 | TTACTACGT***GCGGCCGC***TTATTTCCAGCAAATCAGAC | *Not*I |
| 5’Muta N126N | ATCGCGGCCAATAATTATGACTGGTTCG |  |
| 3’Muta N126N | GTCATAATTATTGGCCGCGATAGCAGAG |  |
| 5’tyr muta 3306-1 | CATGCATGCATGGCACTCCTTTGTGGCTTCGATCCTACCGCTGAC |  |
| 5’tyr muta 3306-2 | GGTAGCCCAGGTGACGGACGAGGAAGCGTTAGCAGAGCGACTGGCGCAAGGCCCGATCGCACTCCTTTGTGGCTTCGA |  |
| Thr37f | CCGATCGCACTCACGTGTGGCTTCGATCCTACCG | - |
| Thr37r | ATCGAAGCCACACGTGAGTGCGATCGGGCCTTGC | - |
| Ala183f | CAGGGTTATTCGGCTGCCTGTGCGAACAAACAGT | - |
| Ala183r | GTTCGCACAGGCAGCCGAATAACCCTGCAACAGG | - |
| Leu186f | TCGGCTGCCTGTCTTAACAAACAGTACGGTGTGG | - |
| Leu186r | GTACTGTTTGTTAAGACAGGCAGCCGAATAACCC | - |
| hSODfp | CATGCATGCATG***AAGCTT***ATGGCGACGAAGGCC | *Hind*III |
| hSODrp | CATGCATGCATG***GAATTC***TCAATGGTGATGGTGATGATGTTGGGCGATCCCAATTAC | *Eco*RI / hexahistidine-tag |
| SODmutf | AAGGTGTAGGGAAGCATTAAAG | - |
| SODmutr | GCTTCCCTACACCTTCACTGGT | - |

All cleavage sites for endonucleases are marked in bold italics; tag-sequences are underlined.

Table S2 continued. DNA primer sequences

| Name | 5’ → 3’ Sequence | cleavage sites / tags |
| --- | --- | --- |
| PGK1fp | GCTTAATGGGGCGCTACAGGGCGCGTGGGGATGATCCACTAGTACTTCAACTCAAGACGCACAGATATTATAACATCTGC | - |
| PGK1rp | CTTAATATTCCCTATAGTGAGTCGTATTACAGCTGTGTTTTATATTTGTTGTAAAAAGTAGATAATTACTTCCTTGATGA | - |
| hSOD1_Strep_fp | AGGAAACGCTGGAAGTCGTTTGGCTTGTGGTGTA ATTGGGATCGCCCAAAGCGCTTGGAGCCACCCGCAG | *Strep*-tag II |
| hSOD1_Strep_rp | CCCTCTAGATGCATGCTCGA***GCGGCCGC***CAGTGTGATGGATATCTGCA***GAATTC***TTATTTTTCGAACTGCGGGTGGCTCC | *Not*I, *Eco*RI / *Strep*-tag II |
| azPheRS1-(6xHis)_fp | TCTTGCAATTGTTTAATCAAGTTACTGCTTGCCATAT GGTGATGGTGATGATGCAT***GAATTC***AGTTGATTGTATGCTTGG | *Eco*RI / hexahistidine-tag |
| azPheRS1-(6xHis)_rp | TTCTGGCAACCAAACCC | - |

All cleavage sites for endonucleases are marked in bold italics; tag-sequences are underlined.
