# Supplementary material for: Performance Analysis of Orthogonal Pairs Designed for an Expanded Eukaryotic Genetic Code
Source: PLoS One. 2012 Apr 6;7(4):e31992. doi: 10.1371/journal.pone.0031992 (PMC3320878; doi:10.1371/journal.pone.0031992)
Supplement: Methods S1 — Contains the technical details for the analysis of the intracellular expression of the TyrRS mutants. (DOC) [file pone.0031992.s014.doc]

# Supporting Information

# Performance analysis of orthogonal pairs designed for an expanded eukaryotic genetic code

## Sebastian Nehring^1^, Nediljko Budisa^1^, Birgit Wiltschi^2,3,4^*

1 Department of Biocatalysis, Technical University of Berlin, Berlin, Germany

2 BIOSS - Centre for Biological Signalling Studies, Albert-Ludwigs-University Freiburg, Freiburg, Germany

2 Faculty of Biology, Albert-Ludwigs-University Freiburg, Freiburg, Germany

4 Present Address: Austrian Centre of Industrial Biotechnology, Graz, Austria

* To whom correspondence should be addressed.
E-mail: birgit.wiltschi@acib.at

**Supplementary Methods**

**Intracellular expression of the TyrRS mutants.** First a C-terminal hexahistidine-tag was added to AzRS1 on pAz1/tRNA_CUA_ by homologous recombination. To achieve this, a DNA fragment encoding the hexahistidine-tag was generated by PCR using primers azPheRS1-(6xHis)_fp and azPheRS1-(6xHis)_rp (see Table S2 for primer sequences). The PCR fragment was flanked by homology hooks which allowed homologous recombination in InvSc1with the pAz1/tRNA_CUA_ vector that had been linearized with *Not*I. The introduction of the hexahistidine-tag coding sequence into the resulting pAz1(His)/tRNA_CUA_ vector was verified by sequencing. For expression studies, the *S.cerevisiae* expression strain InvSc1 was transformed with the modified pAz1(His)/tRNA_CUA_ vector, pAz3/3SUP-tRNA_CUA_, or pPR1/3SUP-tRNA_CUA_. aaRS expression was analyzed in one AzRS1(His) expression clone and three expression clones each for AzRS3 and PxRS1. The cells were grown in 5 mL SC –Trp medium (1% glucose, 0.67% yeast nitrogen base (Difco Laboratories, MI), 1.92 g/L yeast synthetic drop-out medium supplement without tryptophan (Sigma, Deisenhofen, Germany)) at 30 °C for 24 h. Yeast cells were prepared for immunoblotting as described previously [1]. Briefly, a volume of yeast culture corresponding to OD_600nm_ mL = 3 was harvested and the cell pellet was resuspended in 500 µl water. 50 µl 1.85 M NaOH was added and the mixture was incubated on ice for 10 min. Then 50 µl of 50% trichloroacetic acid were added and the mixture was incubated for another 10 min on ice. Precipitates were collected by centrifugation at 12 000 xg for 5 min and the pellets dissolved in 35 µl dissociation buffer (4% sodium dodecyl sulphate, 0.1 M Tris/Cl, pH 6.8, 4 mM EDTA, 20% glycerol, 2% *β*-mercaptoethanol, 0.02% bromphenol blue) and 15 µl 1 M Tris base. After heating at 95 °C for 10 min, 20 µl were loaded onto a 12% SDS gel [2]. After electrophoresis, the proteins were blotted onto nitrocellulose membrane and hexahistidine-tagged aaRSs were detected by a primary monoclonal anti-6x His-Tag antibody from mouse (GTX74966, GeneTex, Irvine, CA) and a horseradish peroxidase-conjugated goat anti-mouse IgG secondary antibody (Bio-Rad, Hercules, CA), followed by chemiluminescence detection (Pierce, Rockford, IL).

1. Riezman H, Hase T, van Loon AP, Grivell LA, Suda K, et al. (1983) Import of proteins into mitochondria: a 70 kilodalton outer membrane protein with a large carboxy-terminal deletion is still transported to the outer membrane. EMBO Journal 2: 2161-2168.

2. Laemmli UK (1970) Cleavage of structural proteins during the assembly of the head of bacteriophage T4. Nature 227: 680-685.
